# Health Workers’ Perspectives on Mobile Health Care Learning Stickiness: Mixed Methods Study

**DOI:** 10.2196/63827

**Published:** 2025-06-13

**Authors:** Sabila Nurwardani, Putu Wuri Handayani

**Affiliations:** 1 Faculty of Computer Science University of Indonesia Depok Indonesia

**Keywords:** mobile health care learning, mobile learning, health worker, stickiness

## Abstract

**Background:**

Doctor-to-Doctor (D2D) is a mobile learning app that aims to support continuous learning in health care, commonly known as continuing medical education. One of the metrics of success in mobile learning is the average amount of time spent each month on the app, which is a component of stickiness, the tendency of users to use apps repeatedly. Stickiness metrics are important because stickiness has a direct effect on user retention.

**Objective:**

This study aimed to determine the factors influencing user stickiness of the D2D mobile learning app. The research framework was based on the stimulus-organism-response theory.

**Methods:**

This study used a mixed methods approach, including a web-based questionnaire (quantitative data) and interviews (qualitative data). We recruited 520 health worker respondents, including general practitioners, dentists, specialists, and medical students, as users of the D2D app. Quantitative data processing was conducted using covariance-based structural equation modeling, whereas qualitative analysis was conducted on the data from 15 respondents using the content analysis method.

**Results:**

On the basis of the web-based questionnaire (quantitative) results, we found that cognitive (*P*=.01) and emotional (*P*=.004) app relationship quality affected health workers’ stickiness in mobile learning. On the other hand, factors related to the functionality of the app and health workers’ experience were proven to affect cognitive and emotional app relationship quality (*P*<.005). In addition, according to interview (qualitative) data, the performance of apps for mobile learning is influenced by information quality and information processing speed, which are needed to deliver a more efficient learning process and reduce the possibility of misunderstanding in the interpretation of learning materials. The user experience is influenced by gamification factors to make the learning process more fun, especially for medical students who do not have to obtain professional credit units (referred to as *satuan kredit profesional* in Indonesia), unlike physicians or specialists.

**Conclusions:**

The results of this study will help mobile learning service providers increase user stickiness in mobile learning, for example, through processing speed, the quality of the information presented, security features, personalized content recommendations, and gamification.

## Introduction

### Background

To date, the health worker and medical student communities have used e-learning and tele-education to enable continuous education and training in the health sector, which is commonly known as continuing medical education (CME) [1]. One form of tele-education is mobile learning [2]. A 2020 report showed that the need to provide CME programs has increased; moreover, the number of medical personnel in Indonesia is always increasing [3]. In 2022, there was an increase in medical personnel in Indonesia by 3.3%, encompassing general practitioners, specialists, dentists, and specialist dentists [4]. Although the number of medical personnel has increased, Indonesia has a ratio of only 0.47 physicians per 1000 population [4]. One of the challenges faced by the health care sector in Indonesia is the uneven distribution of physicians—as many as 71,286 physicians, or approximately 57.63%, are located on the island of Java [5]. This challenge is exacerbated by the geography of Indonesia as an archipelagic country, which makes many physicians reluctant to work in remote areas due to inadequate regional infrastructure [5]. Thus, physicians in regions outside Java face challenges in providing health services and participating in CME programs, which are generally held only in big cities.

In Indonesia, the implementation of a CME program is regulated through Article 28 of Law 29 of 2004, which concerns medical practice. On the basis of that law, all physicians, dentists, and specialists are required to engage in continuous learning via programs conducted by professional organizations on current advancements in science and technology in their respective fields. The implementation of CME includes skill training, webinars, and various events through which attendees earn professional credit units (referred to as *satuan kredit profesional* [SKP] in Indonesia). On the basis of the Decree of the Minister of Health of the Republic of Indonesia HK.01.07/Menkes/1561/2024 concerning the *Guidelines for the Management of the Fulfilment of the Adequacy of Professional Credit Units for Medical Personnel and Health Workers*, SKP points are required to obtain or renew the certificate of expertise, namely, the Registration Certificate issued by the Indonesian Health Workers Council. In Indonesia, physicians and specialist physicians must obtain a score of 250 SKP points for 5 years and dentists and specialist dentists must obtain a score of 100 SKP points for 5 years for clinical (related to direct and indirect medical services) and nonclinical (eg, teaching, researching, conducting health managerial activities, and conducting professional or community service) activities. SKP points are reviewed by the chairman of the Indonesian physician association per region for general practitioners and by the chairman of the association of physicians for specialist physicians.

User stickiness to an app can be considered a key metric for evaluating the relationship between a user and an app [6]. It may influence their decision to adopt or discontinue the use of the app. According to Hsu and Tang [7], the stickiness of a mobile app is its ability to encourage continuous user interaction, which requires that the app maintain users’ interest. Chen et al [8] argue that stickiness is the tendency of users to continue to visit apps or websites. One of the strategies that have been implemented to increase the retention rate (ie, stickiness rate) of apps is reinforcing the desire of the user to use the app. These efforts can take the form of direct promotion through webinars, recommendations and reviews by physicians and medical students, and reminder activity through the notification feature on the app [9].

The Doctor-to-Doctor (D2D) app (PT Global Urban Esensial), which competes with various other apps in Indonesia, including Docquity, Halodoc, and Alomedika, is a pioneer in obtaining SKP points for physicians. These 4 apps are the most popular mobile learning apps in Indonesia, especially regarding support for CME programs and webinars [10]. However, D2D is a pioneer in mobile learning and has high ratings and a large number of users in Indonesia compared to other apps. When developing health-related content for physicians, the materials are curated and validated by the company’s internal medical team, where each piece of content is produced based on current and relevant medical topics [11]. Approximately 88% of D2D users are physicians practicing in Indonesia [11]. Physicians are also encouraged to engage in CME as part of their ongoing professional development. The D2D app offers various webinars tailored to specific target groups, such as webinars specifically designed for pediatricians [11].

**Figure 1:**
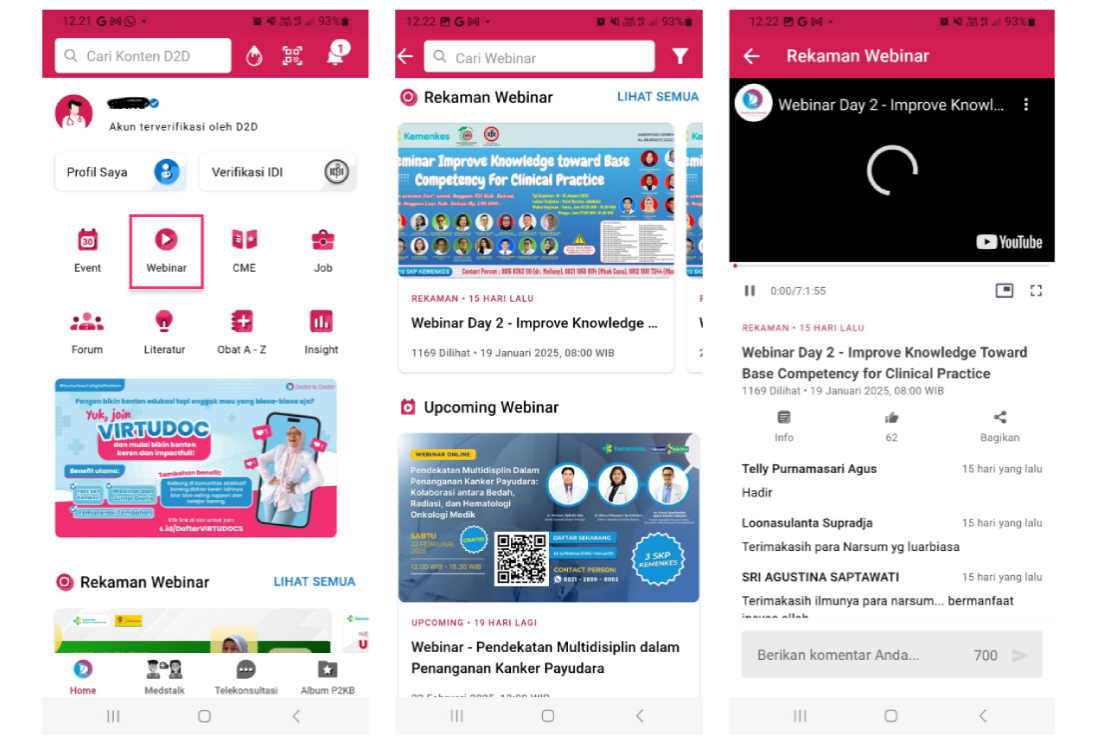
Webinar features of Doctor-to-Doctor.

**Figure 2:**
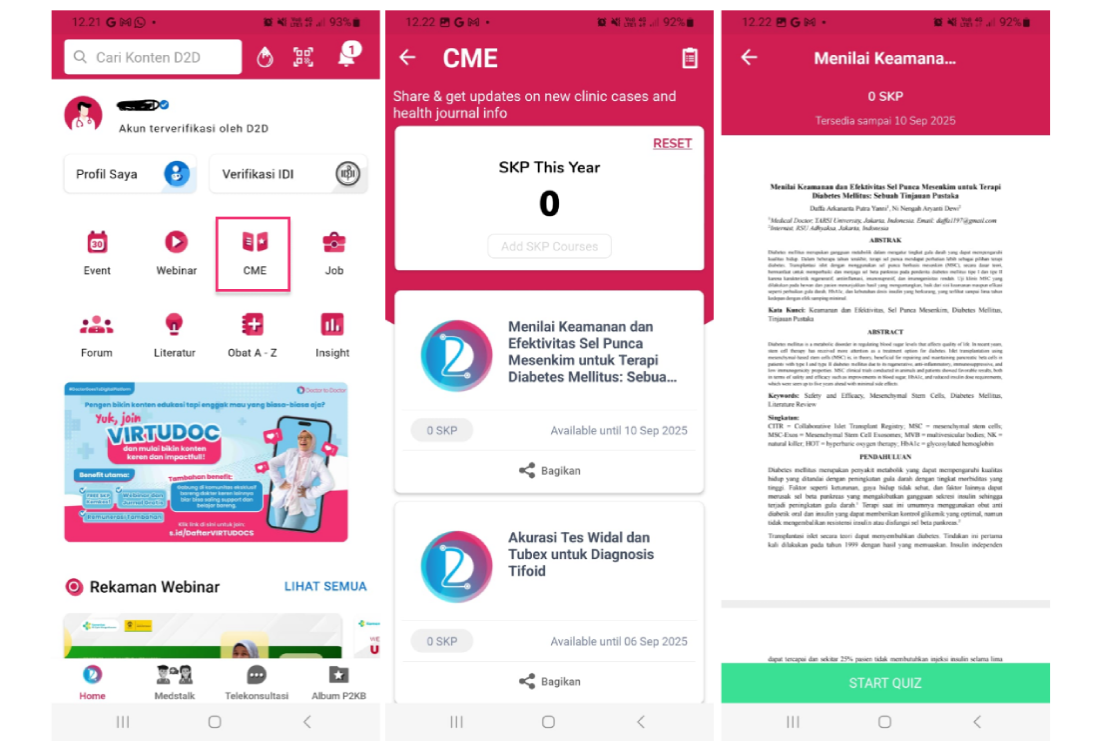
Continuing medical education features of Doctor-to-Doctor.

**Figure 3:**
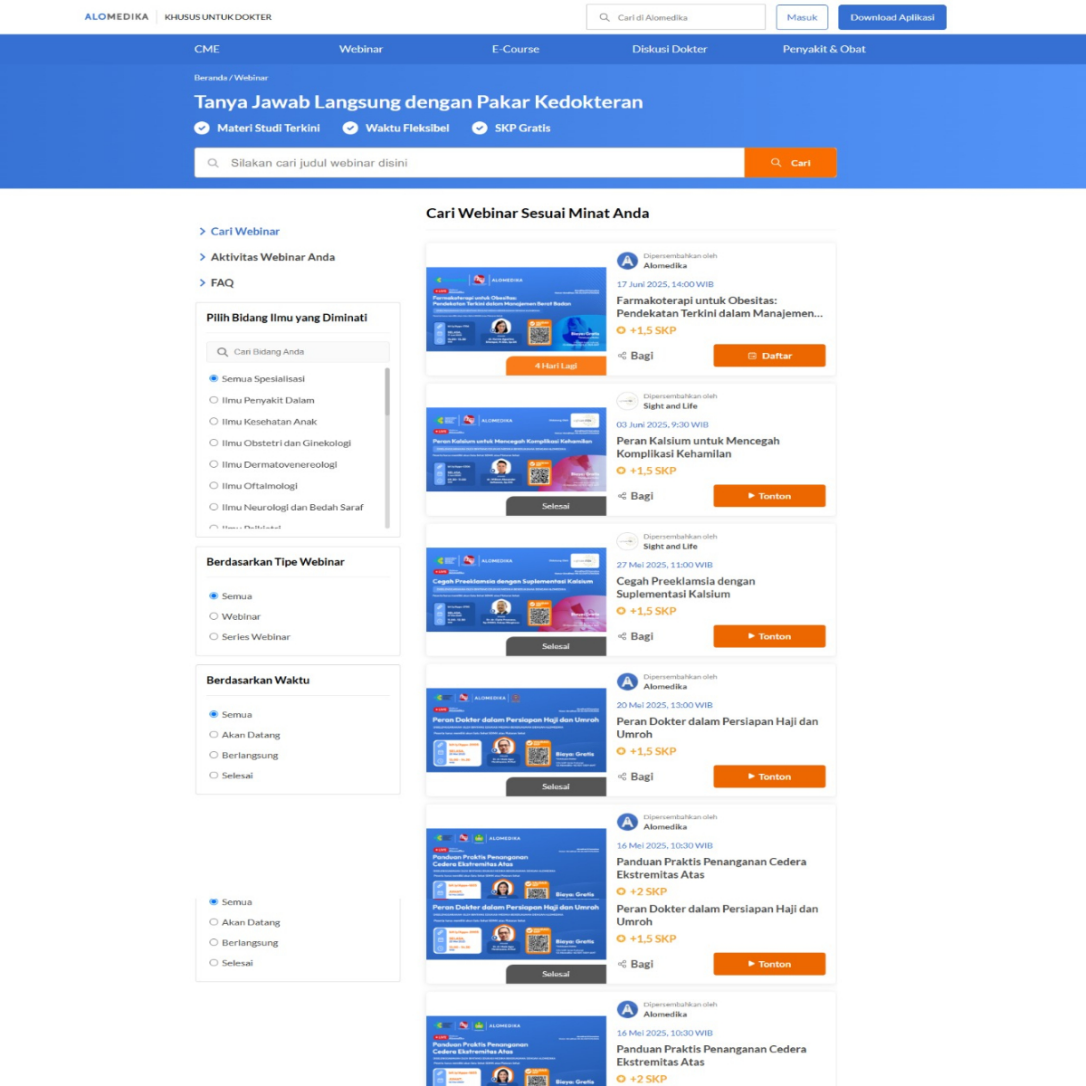
Webinar features of the Doctor-to-Doctor competitor.

The D2D app was first released in 2018, and to date, it already has >80,000 users on the Google Play Store and Apple App Store [11]. In addition, the D2D app is integrated with medical organizations in Indonesia, namely, the Indonesian Medical Association, and has been certified by the Indonesian Ministry of Health as one of the platforms that can be used to obtain SKP points [11]. Additional benefits for D2D users include access to the latest health content and information, up-to-date information on medical events, and the opportunity to attend free and certified webinars. Features of the D2D app include access to free webinars, health journals, medical discussion forums, and loyalty programs. However, the results of the study by Halim et al [12] show that D2D is only the fourth most frequently accessed app by physicians and medical students. Even though D2D is a pioneer in mobile learning and has amassed high ratings and a large number of users in Indonesia, this does not guarantee that the app will have a high level of user attachment or stickiness. Figures 1 and 2 show example webinar and CME features, respectively. The D2D app’s health content is not limited to the D2D health worker community but can also be accessed by all health workers, which is not the case for its competitors such as Alomedika, as shown in Figure 3.

### Objectives

In the context of e-learning, stickiness can also be affected by the emotions of the users when using an app, such as those that drive a user’s decision to continue using or delete an app [13,14]. Moreover, the system performance of the mobile learning app plays an important role in user attachment, specifically in meeting user needs [9]. Thus, stickiness, which is related to both the user’s emotions and the performance of the app, is an important benchmark for measuring the intensity of mobile learning use. However, research related to user stickiness is still focused on retail apps [6,15], massive open online courses [16-18], and fitness apps [9]. Therefore, this research sought to answer the following question: What factors affect health workers’ stickiness regarding mobile learning apps? We used a web-based questionnaire and interviews to reach a large number of respondents and triangulated quantitative results with interview data to better understand the findings of this study. We formed the hypotheses underlying this study by analyzing the relationship between functional and experiential factors that could influence cognitive app relationship quality (CARQ) and emotional app relationship quality (EARQ), which influence app stickiness among users of mobile learning apps. The results of this study can provide guidance for mobile learning service providers to formulate strategies for improving stickiness rates.

## Methods

### Research Model

**Figure 4:**
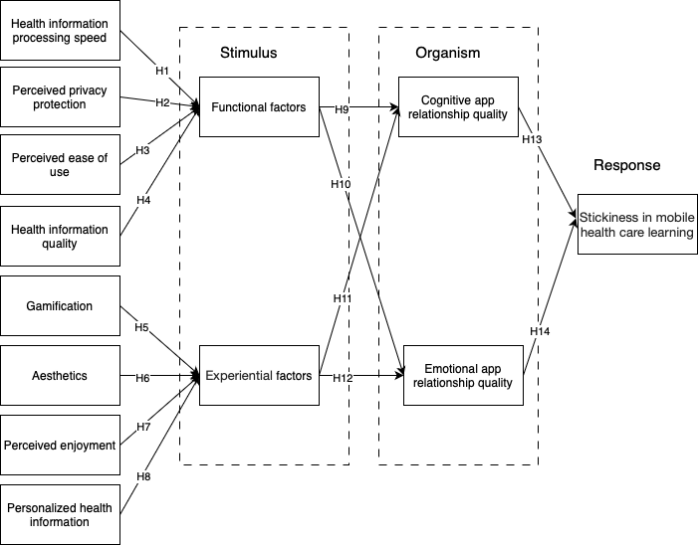
The proposed research model. H1 to H14: hypothesis 1 to hypothesis 14.

This study’s research model was based on the stimulus-organism-response theory [19] that explains the influence of a stimulus on the user assessment process and the relationship between the results of the individual assessment process and user habits [19]. The design of the proposed model is referred to in multiple studies [6,13,20,21]. The selection of factors was adjusted to the scope of this study, namely, factors that affect user behavior regarding continuous use of an app (ie, user stickiness). This research focused on the theory of app relationship quality, namely, CARQ and EARQ, where the quality of the app relationship is driven by functional and emotional factors, respectively. Figure 4 shows the research model used in this study.

In this study, health information processing speed (HIPS) refers to the level of information processing speed in a given system [6]. This constitutes the ability of the system to process information, load pages, display content, or perform other functions [15]. The system speed is the speed at which a system can access information via an access link [22]. When an app can process user input or requests quickly and is able to present results without a delay, it becomes more convenient and efficient for users to carry out activities on the app [23]. Therefore, it can be concluded that the app response speed is one of the most important functional criteria for users of an information system. Indeed, Alnawas et al [6] mention that speed is one of the factors that affect product functionality. Thus, we propose the following hypothesis: HIPS influences functional factors in mobile learning performance assessment (hypothesis 1).

Perceived privacy protection (PPP) is defined as an app’s ability to protect users’ personal data [24]. This includes protection of data related to the users’ browsing history and transaction history and other personal information [24]. On the other hand, PPP is also part of the security dimension of an app [6], and it can include the extent to which an app is able to protect users’ personal data from unauthorized access by other parties [25]. Implementation of PPP can include notifying users that their personal data will not be shared with other parties [26]. Such implementation is also critical to preventing data problems caused by the unauthorized use of personal data [27]. Therefore, we propose the following hypothesis: PPP influences functional factors in mobile learning performance assessment (hypothesis 2).

Perceived ease of use (PEU) is defined as a user’s assessment of an app based on its ease of use or performance [6]. PEU can also refer to the level of convenience that users feel when operating the app, such as not being confused when using or searching for information on the app for the first time [7]. The convenience that users feel will have a direct effect on their perception of the benefits obtained from the app [28]. The easier an app is to use, the greater the incentive for users to continue carrying out activities without feeling confused about the appearance or features [23]. Ease of use also reduces the time it takes for users to understand the app and its functionality and find solutions when facing problems with the app [25]. Previous research has shown that PEU has an influence on user satisfaction and stickiness [29]. This is also supported by the work by Hsu and Tang [7], who included PEU in the top 5 factors considered to have the most influence on the user experience ratings of retail apps. PEU is also related to the functionality of an app as users prefer that an app be easy to operate—the app should be pleasing to use and should provide the requested information quickly and accurately [6]. Therefore, we propose the following hypothesis: PEU influences functional factors in mobile learning performance assessment (hypothesis 3).

Health information quality (HIQ) is defined as the level of information quality on an app, including the accuracy, reliability, clarity, and relevance of the content [29]. Such quality can be measured based on the speed at which the user reads the information and on the richness and reliability of the content (trusted sources) [29]. The content presented on an app must be informative and provide new insights to users [12]. Cognitive judgment is influenced by product functionality, an aspect of which is the ease with which users can find information [21]. In the context of mobile learning research, information is obviously a very important element in user learning [8]. The perceived usefulness (utilitarian benefit) of the information obtained during the learning process is the main focus of users of a given app [8]. Therefore, we formulate the following hypothesis: HIQ influences functional factors in mobile learning performance assessment (hypothesis 4).

Gamification is the implementation of game features in an app to increase motivation and interaction between users [13]. It can also refer to the adoption of a certain game design to influence self-awareness and individual behavior during the use of an app [13]. Gamification includes elements such as rewards or challenges that motivate users to continue interacting with an app [13]. Yang and Li [30] described the influence that gamification has on the user’s experience and their decision to continue using a given mobile learning app. Aparicio et al [16] have similarly shown that gamification and personalization have a positive influence on the user app experience in massive open online courses. In the field of education, gamification features can encourage learners to become more involved in the learning process [16]. The adoption of gamification has been proven to encourage users to engage in more web-based learning activities, provide an enjoyable learning experience, and stimulate users to learn more [16,31]. Djohan et al [32] found that gamification also influences the user experience of an app. Hence, we propose the following hypothesis: gamification influences the experiential factors of the user’s perceived experience of mobile learning (hypothesis 5).

Aesthetics, for the purposes of this study, are a perception produced by the visual appeal of an app through the selection of colors and illustrations, among other things [28]. Through the experience of visual beauty, aesthetics are indirectly able to attract the attention of users [33]. Aesthetics also provide immediate pleasure that is unrelated to the app’s functionality or performance assessment [6]. According to Huang et al [33], users feel pleasure when they observe a beautiful app design display. What constitutes a beautiful display is influenced by a number of factors, including strategically arranged feature layouts, easy-to-reach search bar placement, feature placement design based on user need priorities, the use of unobtrusive colors and attractive icons, and the effective use of typography [24]. Huang et al [33] showed that the aesthetic value of an app has a significant influence on user experience. These findings are also supported by the work by Rose et al [34], who found that the aesthetic value of an app’s display can affect the user’s emotions while using the app. Therefore, we propose the following hypothesis: aesthetics influence the experiential factors of the user’s perceived experience of mobile learning (hypothesis 6).

Perceived enjoyment (PE) is the user’s perception of feeling happy or entertained or of enjoying the content or features provided by an app [7]. PE tends to be related to the user’s emotions (eg, pleasure, happiness, and being entertained) while they are using the system. Pleasure or satisfaction in using an app is not determined by system performance alone but is more often influenced by the overall user experience [35]. On the basis of the work by Hsu and Tang [7], PE is one of the top 5 most important factors in creating a sticky app. The enjoyment that users feel can also increase interactivity when using an app [7]. Thus, we propose the following hypothesis: PE influences the experiential factors of the user’s perceived experience of mobile learning (hypothesis 7).

Personalized health information (PHI) is a part of information personalization, the automatic provision of content based on user preferences and certain classifications [6,7]. Personalization refers to the app’s ability to offer customized information, services, design, and content to meet user needs and preferences [36]. This helps users focus on what they want, determine the fitness of a product or service, and complete tasks efficiently [37]. Personalization provides customized content to improve the quality of the provided information and encourage users to complete certain tasks; personalization also encompasses user comfort [38]. The benefits offered by the adoption of personalization include the enjoyment that users feel [39], positive emotions [38], and feelings of joy [40]. Alnawas et al [6] found that personalization has a significant influence on the user experience. This is supported by the work by Cheng [13], who indicated that information personalization can affect the user experience and significantly affect the user’s habit of using the app. Thus, we propose the following hypothesis: PHI influences the experiential factors of the user’s perceived experience of mobile learning (hypothesis 8).

Functional factors are factors that directly affect the success of an app’s performance [6]. Functionality is the main factor in evaluating the usefulness (ie, utilitarian benefit) of an app [41]. This is because app functionality influences the app’s ability to help users achieve their goals safely and efficiently [6,41]. Cognitive assessment, the result of a comparison of expectations with app capabilities, can be conducted based on app functionality [7]. Wu [42] found that the main factor that significantly influences user engagement with an app is users’ performance expectations. Therefore, we propose the following hypothesis: functional factors influence CARQ in the context of user assessment of the perceived usefulness of mobile learning (hypothesis 9).

Al-Nabhani et al [43] noted that functional factors also influence user satisfaction, which significantly affects users’ emotions. The functionality of the app is another factor that users feel is related to their sense of pleasure or satisfaction with the app [44]. Responses related to the perception of emotions are called “judgment affective” responses and are related to EARQ [6]. Alnawas et al [6] state that a user’s assessment of an app’s functionality affects the emotional impact of a retail app, although not as strongly as its cognitive impact. Similarly, Molinillo et al [21] found that system functionality influences affective experience when using an app. Therefore, we propose the following hypothesis: functional factors influence EARQ in the context of user assessment of mobile learning based on emotional perception (hypothesis 10).

Experiential factors are defined as factors related to the user’s subjective experience, including aspects such as perception, emotion, preferences, and user interaction with a product [6]. User experience can affect the sensation of using the app [23]. This refers to the psychological state experienced by users when evaluating the app, that is, the emotions felt while using the app [6]. Customer experience is subjective and contextual because it can differ among customers, cultures, and situations [32]. Each individual may have different feelings because their experience affects their assessment of the app [32]. Any emotions that users experience using an app generally affect the perception of pleasure and can encourage users to continue using the app [45]. Although user experience is not directly related to app performance, it affects the user’s assessment of the usefulness of the system [6]. In this case, emotions become an important part of cognitive assessment, specifically related to processing the value of an experience, and can form the basis for a deep and intense relationship with the app [6]. User experience also significantly influences satisfaction and trust, to the point that experiences can form the basis for an emotional connection and a deep interest in a given product [6]. Thus, we suggest the following hypothesis: experiential factors influence CARQ in the context of user assessment of the perceived usefulness of mobile learning (hypothesis 11).

Experiential factors include users’ emotions in relation to their assessment of the usefulness of an app [6]. Indeed, the emotions felt by users occur and are processed through human senses, especially those related to the appearance of the app [38]. User experience is the experience of users when they do something that involves them psychologically in activities requiring concentration, motivation, and enthusiasm [6,32]. This user experience includes aesthetic value, perception of pleasure (ie, enjoyment), and gamification [6,46]. Moreover, van Noort and van Reijmersdal [45] found that, if an app offers entertainment, it can produce a fairly good level of app performance satisfaction based on the user’s emotional perception. User experience, derived from users’ emotional assessments of a technology, plays a critical role in determining the use and benefits of the technology, including in the context of a mobile app [7]. Thus, we propose the following hypothesis: experiential factors influence EARQ in the context of user assessment of mobile learning based on emotional perception (hypothesis 12).

Alnawas et al [6] described how EARQ and CARQ affect the level of attachment of app users (ie, stickiness). Zolkepli et al [44] explained that this effect is related to differentiating user behavior based on feelings (affective) and thoughts (cognitive). Cognitive assessment is based on rational thinking, so it is more changeable and vulnerable to external influences such as other people’s opinions [47]. The cognitive assessment of apps is usually related to the ease with which users can find factual information in accordance with their needs [48]. A relationship based on CARQ emphasizes analysis of the benefits obtained by the user, so it is necessary to conduct periodic evaluations of the usefulness of the available features [47]. In addition, it has been proven that user satisfaction with app performance—in the context of utilitarian benefits—positively affects the intensity of use of mobile apps [6]. Thus, both the quality of the app’s performance and its emotional approach to the user experience can influence the user’s desire to invest time and effort in using the app [49]. Therefore, we propose the following hypothesis: CARQ influences stickiness in mobile learning apps and determines the intensity of daily use (hypothesis 13).

Behaviors based on feelings tend to be more subjective because they relate to aspects of positive or negative judgment, whereas behaviors based on thoughts tend to be more objective and relate to notions of right or wrong [44]. As responses that arise due to feelings or emotions tend to be more subjective, it is quite difficult to change affective perceptions [21]. Emotional perceptions experienced while using mobile learning apps greatly affects app access intensity during the web-based learning process [13]. Perceptions that increase the duration of app access generally tend to be positive, such as feeling happy and entertained and enjoying using the app [50]. Nyffenegger et al [51] stated that user attachment to an app can be reflected through satisfaction and trust built based on the intensity of app use. User engagement with the app is the result of user assessments based on experience (ie, affective experience) [21]. Affective judgment is judgment based on the feelings of an individual, which makes influence by external forces more difficult [48]. Decisions based on experience are difficult to change as they shape individual beliefs and perceptions, including the use of apps [48]. Fernandes and Proença [47] explained that, when attitudes are dominated by feelings, individuals tend to behave habitually, making those feelings resistant to change and resulting in a much stronger response compared to attitudes based purely on rational thinking. Thus, we propose the following hypothesis: EARQ influences stickiness in mobile learning apps and determines the intensity of daily use (hypothesis 14).

### Research Process

We used a quantitative approach to test the hypotheses and a qualitative approach to more deeply analyze the results of the hypothesis testing. An online questionnaire was used as a quantitative research instrument, and interviews were used to collect qualitative data. The focus of this study was on mobile learning, namely, the D2D app. We used purposive sampling, where all respondents involved in this study were D2D users—physicians, dentists, specialists, and medical students (eg, coassistants, residents, dental students, and medical undergraduate students). Furthermore, we asked the D2D company to help distribute the questionnaire links through push notifications on the D2D app. We also used social media platforms such as WhatsApp, X (formerly known as Twitter), and Instagram, which are popular in Indonesia, to reach more health care workers.

We conducted a readability test on the questionnaire to ensure that the research instrument was easily understandable, in accordance with writing standards, and relevant to the context of the research. The readability test was carried out from February 1, 2024, to February 7, 2024. In total, 6 respondents participated in the readability test process. Most of the changes after the readability test were made due to ambiguous word choice or unclear syntax. After the readability test, a pilot study was conducted to test the reliability of the research instrument.

The pilot study was conducted to find whether participants experienced problems filling out the questionnaire before it was disseminated to more participants. The pilot study was conducted from February 19, 2024, to February 23, 2024, with a total of 50 respondents. The total Cronbach α value obtained in the pilot study was 0.981; thus, we distributed the questionnaire link to a larger number of respondents. Finally, based on the results of the quantitative analysis, hypotheses 2, 3, 6, 7, 8, and 12 were rejected. We used a qualitative instrument (semistructured interviews) to determine the reason for these rejections and obtain a deeper understanding of the results. Online and offline interviews were conducted with 15 respondents.

### Research Instruments

The web-based questionnaire included demographic questions and measurement items. The preparation of the measurement items was carried out with reference to previous research, such as the studies by Alnawas et al [6], Chen et al [8], Cheng [13], Hsu and Chen [52], Huang et al [33], Elsotouhy et al [9], and Yang et al [53], and contextualized for this study, namely, mobile learning in health care. To make it easier for respondents to fill out the questionnaire, we translated the measurement items in Indonesian and tested them by conducting readability tests and pilot studies to ensure that respondents could understand the questionnaire. Each measurement item was given a code according to the variables it represented to make it easier to process the data. The assessment of the measurement items was then carried out using a 5-point Likert scale that included options ranging from “strongly disagree” to “strongly agree.” Multimedia Appendix 1 contains the questionnaire instrument and the 13 interview questions. We defined the interview questions according to the variables used in this study. The results of the questionnaire were analyzed using covariance-based structural equation modeling in SPSS Amos (version 24; IBM Corp).

In addition, the qualitative data were analyzed using content analysis. The results of the semistructured interviews were interpreted and linked to the hypotheses identified during the quantitative data analysis stage. The qualitative codification process was carried out using interview quotes that were grouped according to the specific hypotheses. Multimedia Appendix 1 provides the details of the qualitative results. An example of the codification results can be found in the Qualitative Results and Validity of Hypothesis Testing section.

### Ethical Considerations

This study received approval from the research unit of the Faculty of Computer Science, University of Indonesia (reference S-17/UN2.F11.D1.5/PPM.00.00/2024). In line with university policy, the Research and Community Service Department, Faculty of Computer Science, University of Indonesia, adhered to the guidelines and procedures established by the faculty and provided ethics approval for this study. This study was also approved by the D2D company (reference 761/GUE/IX/2024/E). All respondent data were anonymized, all questionnaire respondents provided written informed consent, and all the interview participants provided verbal informed consent to take part in this study. Participants did not receive compensation.

## Results

### Quantitative Results

#### Respondents’ Demographics

Data collection for the primary study was carried out for approximately 25 days, from February 29, 2024, to March 21, 2024. The number of respondents who filled out the complete questionnaire was 520, which fulfills the requirements by Hair et al [54], where the minimum number of respondents required is the number of measurement items multiplied by 10. Detailed respondent demographic characteristics are shown in Table 1, and Table 2 shows the demographic characteristics of the interviewees. The interviews lasted between 30 and 60 minutes and took place between April 19, 2024, and April 27, 2024.

**Table 1:** Questionnaire respondents’ demographic characteristics (N=520).

Variable			Respondents, n (%)
**Gender**			
	Woman	311 (59.8)	
	Man	209 (40.2)	
**Age (y)**			
	<20	3 (0.6)	
	20-30	206 (39.6)	
	31-40	132 (25.4)	
	41-50	96 (18.5)	
	>50	83 (16)	
**Domicile**			
	Greater Jakarta	280 (53.8)	
	Java island outside Greater Jakarta	103 (19.8)	
	Outside Java island	137 (26.3)	
**Occupation**			
	General practitioner	358 (68.8)	
	Specialist	59 (11.3)	
	Dentist	4 (0.8)	
	Coassistant	46 (8.8)	
	Resident	7 (1.3)	
	Dental student	6 (1.2)	
	Medical undergraduate student	40 (7.7)	
**D2D^a^ app use period (mo)**			
	<6	97 (18.7)	
	6-12	94 (18.1)	
	13-24	131 (25.2)	
	>24	198 (38.1)	

^a^D2D: Doctor-to-Doctor.

**Table 2:** Summary of the interviewees’ demographic characteristics.

Respondent	Gender	Age (y)	Occupation
1	Woman	23	General practitioner
2	Woman	25	General practitioner
3	Woman	23	Dentist coassistant
4	Man	23	Coassistant
5	Woman	19	Medical student
6	Man	24	General practitioner
7	Woman	23	General practitioner
8	Woman	23	Dentist coassistant
9	Man	25	General practitioner
10	Woman	20	Medical student
11	Woman	24	General practitioner
12	Woman	36	General practitioner
13	Woman	24	General practitioner
14	Man	19	Medical student
15	Man	19	Medical student

#### Measurement and Structural Model Testing

The average variance extracted (AVE) value is obtained from the sum of the squares of the values of the factor loadings of each indicator divided by the sum of the squares of the values of the factor loadings of each indicator, and this is added to the total measurement error of all indicators on one variable [55]. The AVE value of a variable is declared valid if it is >0.5 [54,55]. On the basis of the results, all variables in the research model passed the AVE value test (Table 3). The reliability test stage was carried out by checking the value of the composite reliability on each latent variable. The composite reliability of each latent variable is said to be valid if it has a value of >0.7 [54]. On the basis of the results shown in Table 3, all latent variables in this study passed the reliability test.

**Table 3:** Average variance extracted (AVE) and composite reliability (CR) test results.

Variables	CR	AVE
Stickiness in mobile learning	0.736	0.583
Cognitive app relationship quality	0.79	0.654
Emotional app relationship quality	0.991	0.983
Functional factors	0.99	0.979
Experiential factors	0.752	0.603
Health information processing speed	0.718	0.561
Perceived privacy protection	0.838	0.72
Perceived ease of use	0.989	0.979
Health information quality	0.739	0.588
Gamification	0.802	0.67
Aesthetics	0.849	0.585
Perceived enjoyment	0.814	0.687
Personalized health information	0.857	0.601

#### Hypothesis Testing

After the research model passed the validity and reliability tests, the next step was to check the goodness of fit (GOF) of the model. The GOF test consists of absolute fit indexes, incremental fit indexes, and parsimony fit indexes. Absolute fit indexes determine model compatibility by checking the values of the root mean square residual, GOF index, adjusted GOF index, and root mean square error of approximation. Incremental fit indexes are tests of normed fit index, comparative fit index, and Tucker-Lewis index values. Meanwhile, parsimony fit indexes test the parsimonious normed fit index. The results of the GOF test are shown in Table 4.

The determination coefficient test was carried out to determine how well the endogenous variables simultaneously explained the exogenous variables [54]. The determination coefficient, which ranges from 0 to 1, is calculated from the square of the correlation (*R*^2^) between its dependent and independent variables [54]. The *R*^2^ value is categorized as strong if it is >0.67, moderate if it is between 0.33 and 0.67, and weak if it is >0.19 but <0.33 [54]. The results of the determination coefficient test are shown in Table 5.

In this study, we used a 2-tailed hypothesis test. If the *P* value was <.05, then the hypothesis was accepted; if the *P* value was >.05, the hypothesis was rejected [54]. The results of the hypothesis test are shown in Table 6.

**Table 4:** Goodness-of-fit test results.

Test	Requirement	Result	Description
Chi-square	>0.05	315.5	Good fit
Chi-square divided by *df*	<2.0	1.024	Good fit
Goodness-of-fit index	>0.9	0.949	Good fit
Root mean square error of approximation	≤0.08	0.008	Good fit
Root mean square residual	≤0.05	0.02	Good fit
Normed fit index	≥0.9	0.943	Good fit
Relative fit index	≥0.9	0.92	Good fit
Tucker-Lewis index	≥0.9	0.998	Good fit
Comparative fit index	≥0.9	0.999	Good fit
Parsimonious normed fit index	0-1	0.668	Good fit
Adjusted goodness-of-fit index	≥0.9	0.923	Good fit

**Table 5:** Results of the determination coefficient test (R2).

Variables	*R*^2^ value	Description
Experiential factors	0.342	Moderate
Functional factors	0.201	Weak
Emotional app relationship quality	0.170	Weak
Cognitive app relationship quality	0.287	Weak
Stickiness in mobile learning	0.421	Moderate

**Table 6:** Hypothesis test results. The arrows represent the direction of influence.

Hypothesis	Estimate	*P* value	Result
Hypothesis 1: HIPS^a^ →FFs^b^	0.282	.01	Accepted
Hypothesis 2: PPP^c^→FFs	0.055	.58	Rejected
Hypothesis 3: PEU^d^→FFs	−0.036	.49	Rejected
Hypothesis 4: HIQ^e^→FFs	0.177	.04	Accepted
Hypothesis 5: GM^f^→EFs^g^	0.337	.005	Accepted
Hypothesis 6: AE^h^→EFs	0.214	.07	Rejected
Hypothesis 7: PE^i^→EFs	0.081	.43	Rejected
Hypothesis 8: PHI^j^→EFs	0.071	.55	Rejected
Hypothesis 9: FFs→CARQ^k^	0.343	.006	Accepted
Hypothesis 10: FFs→EARQ^l^	0.505	.003	Accepted
Hypothesis 11: EFs→CARQ	0.143	.003	Accepted
Hypothesis 12: EFs→EARQ	0.102	.11	Rejected
Hypothesis 13: CARQ→SHL^m^	0.412	.01	Accepted
Hypothesis 14: EARQ→SHL	0.416	.004	Accepted

^a^HIPS: health information processing speed.

^b^FF: functional factor.

^c^PPP: perceived privacy protection.

^d^PEU: perceived ease of use.

^e^HIQ: health information quality.

^f^GM: gamification.

^g^EF: experiential factor.

^h^AE: aesthetics.

^i^PE: perceived enjoyment.

^j^PHI: personalized health information.

^k^EARQ: emotional app relationship quality.

^l^CARQ: cognitive app relationship quality.

^m^SHL: stickiness in mobile learning.

### Qualitative Results and Validity of Hypothesis Testing

#### Hypothesis 1: HIPS and Functional Factors

This study showed that health information processing has an influence on functional factors (hypothesis 1). This is in accordance with the results of previous research related to the effect of stickiness on retail mobile apps, where information processing speed significantly affects app performance (functional factor) [6]. This is also supported by the work by Hsu and Tang [7], who found that response speed in an app is an important indicator of app functionality in terms of responsiveness. In health app studies, speed has an influence on the user’s attachment and decision to continue using those apps [9]. In the context of mobile learning by medical students, the speed of information processing plays an important role in facilitating a more efficient learning process, allowing students to receive information faster, thereby reducing the possibility of misunderstandings in the interpretation of learning materials [1].

The results of the interviews showed that interviewees agreed that the speed of information processing was an important part of the functionality of the app—the app should be fast and meet user expectations:

The response speed of the app is very good and fast. There are no obstacles in accessing all the features in the D2D app.

Respondent 1

On the other hand, users were also happy if the presentation of the information was fast and occurred without advertisements:

Yes, it has to be fast and not load without ads.

Respondent 4

However, from the users’ perspective, there were network constraints that sometimes made it difficult to access information or content on the mobile learning app:

...Never have experienced a loading failure on the app page or GT error. Most of all, what I have told you is that the network in my area is bad.

Respondent 2

#### Hypothesis 2: PPP and Functional Factors

PPP did not influence functional factors in this study (hypothesis 2). According to the *Harvard Business Review* [56], PPP is not a priority for users—because users are in a hurry to use the app, they do not pay much attention to the security aspect of personal data [56]. On the basis of the results of the interviews, there is a growing sense that users’ trust in the app’s ability to keep personal data secure does not affect their assessment of the app’s functionality:

...Honestly, the functionality is more of a process to create a password and verify it is not complicated, which makes it easier for users. For data security, it’s more about creating a sense of trust in users.

Respondent 13

Regarding the protection of personal data, users trusted the app more when it partnered with a trusted organization than when the capabilities of the security system were invisible in the user interface:

As long as the app is already partnering with a trusted organization.

Respondent 8

However, users also wanted the appearance and flow of the app to remain easy to understand even when there was a process of verification and password reset, which is generally considered to require more steps for security:

I think yes, the more requests the better. But on the other hand, I became very lazy because the process was too complicated.

Respondent 4

PPP plays a greater role in influencing user trust in the app. Although keeping personal data secure often involves a time-consuming process, users preferred simpler and faster verification processes and security settings.

#### Hypothesis 3: PEU and Functional Factors

In contrast with the study by Alnawas et al [6], this study showed that PEU has no significant effect on functional factors (hypothesis 3). This hypothesis was rejected because users are often not proficient in using the information system provided by the app. This finding is supported by respondents’ answers regarding the main obstacles they encountered when accessing the app, namely, time limitations and a busy practice schedule, which were mentioned by 69.4% (361/520) of the respondents. According to Smith [57], PEU also has no effect on the assessment of system performance because users find it difficult to locate information that matches their preferences. This is further supported by the work by Rakhmadian et al [58], who found that users become unhappy if they only have limited access to the information provided by the system. According to the findings of the interviews, users experienced constraints and limitations in feature functionality when using mobile learning. First, users found it difficult to navigate the account verification and registration processes. Second, users often felt that notifications that suddenly appeared during the use of the app were somewhat annoying:

Sudden updates are often confusing.

Respondent 10

Third, users found it difficult to begin working within the app because there were often sudden account log-ins and exits that forced them to re-enter their medical ID number:

I have trouble quite often. Suddenly, go out and go in again. Suddenly, log out and log in to the account again.

Respondent 12

#### Hypothesis 4: HIQ and Functional Factors

The results of this study showed that HIQ has an influence on functional factors (hypothesis 4). This is also supported by the work by Elsotouhy et al [9], who stated that information quality, which is the result of a comparison between expectations and user perceptions of the presented information, is an indicator of functional factors. The quality of the information also determines the adequacy, relevance, thoroughness, and timeliness of user interpretation [59]. In a study of user stickiness regarding mobile news apps, it was found that the information quality affects the satisfaction and attachment of app users [29].

According to the interviewees, the existence of reliable references and sources can minimize misunderstandings or the spread of hoaxes:

...minimizing misunderstandings of hoax information because the source of information is not detailed and clear.

Respondent 2

Interview respondents were also happy with the information presented via D2D because it included references, clear titles, trusted speakers, and rewards such as certificates after completing a webinar. However, some interviewees mentioned shortcomings regarding the content presented on the app (eg, the health journal content was not as complete as on the journal’s website: “...less complete than those available on the journal website” [Respondent 1]). In addition, it was also rare to find health articles written by physicians on the app:

Unfortunately in D2D, not all doctors are authors. Unfortunately, there are no results of doctors’ research in D2D.

Respondent 5

This is in line with the work by Yang et al [53], who noted that the quality of the information affects system performance and user stickiness.

#### Hypothesis 5: Gamification and Experiential Factors

This study found that gamification has an influence on experiential factors (hypothesis 5). This result is in line with the work by Cheng [13], who described how gamification plays a role in the learning process and significantly affects the user experience, thus impacting emotional attachment to an app (EARQ). Gamification features of web-based learning include reward elements and leaderboards to encourage learners to participate [24]. In research related to stickiness in retail apps, gamification is an important element in attracting users and retaining app use [7]. Another study found that gamification has a high influence on experiential factors in mobile commerce apps [32].

The results of the interviews showed that gamification made using the app more fun:

It feels like a learning app with a gamification feature makes it fun and exciting. The collection of program points or SKP points is one of the motivations for me to access webinars or learn apps.

Respondent 1

However, it was unfortunate that the gamification in the D2D app focused more on the SKP point collection program. This did not incentivize users with medical student or dental student profiles to join the app program because they had no need to obtain SKP points:

Never made, because there is no need yet.

Respondent 3

In addition, physicians who still have internship status even though they have obtained a registration certificate as a physician cannot participate in the program:

Currently, there is no need to use SKP points because they are still using Internship [points].

Respondent 9

Even so, gamification is an interesting feature of this mobile learning app because it presents a dashboard that is significantly more informative than that of its competitors:

I think this is one of the main factors for using D2D. Unlike the next app, which only tracks the number of SKP points—it cannot collect SKP points.

Respondent 6

#### Hypothesis 6: Aesthetics and Experiential Factors

This study found that aesthetics have no significant effect on experiential factors (hypothesis 6). This result is in contrast to those of Alnawas et al [6], who found a significant relationship between aesthetics and experiential factors in retail apps. Zhou [59], whose results also contrasted with those of Alnawas et al [6], noted that design trends are dynamic, which affects users’ aesthetic preferences regarding a website’s (also dynamic) appearance. This is also supported by the work by Chen et al [8]—an increased number of target users of an app can affect subjective judgments related to the appearance of the app interface. As demonstrated by the demographic results, this mobile learning app has a diverse target user pool that includes general practitioners, specialists, dentists, medical students, dental students, coassistants, and residents. Thus, hypothesis 6 was rejected due to the variety of app user personas, which leads to many subjective judgments related to the visual assessment of the app*.*

Most interviewees stated that, when making decisions about using a product, the visual appearance or aesthetic aspect of the product is an important factor that is based on subjective interest:

I think it’s very important, if I am not attracted to it from the beginning, I’m lazy to open it again.

Respondent 5

However, the interviews also showed that the color selection in the mobile learning app is still too striking:

...don’t use the color of the ring.

Respondent 2

In addition, it was found that the text displayed by the mobile learning app was too small, so it was difficult to read:

The selection of the size of the text is too small.

Respondent 15

Moreover, some users suggested emphasizing the app’s superior features, namely, medical discussions, so that users are more interested in participating in them:

If possible, the superior features that are emphasized in the medical discussion so that it can be more interactive between users.

Respondent 12

Other users suggested adding animation elements, such as a mascot, to create a lively atmosphere on the app:

My suggestion is adding a mascot or other animation to make it interesting.

Respondent 5

Finally, users indicated that they expected periodic design improvements to prevent them from getting bored with the appearance:

...The design is also always updated so that you don’t get bored.

Respondent 15

The interviews indicated that there are many possible improvements to the appearance of the app that the development team can consider. This reinforces the statement by Zhou [59] that it is necessary to continuously improve the appearance of an app. The appearance of the mobile learning interface should also be tailored to user preferences [17]. Recommendations for mobile learning service providers include evaluating the app design using more comprehensive methods such as heuristic evaluation or user experience design.

#### Hypothesis 7: PE and Experiential Factors

PE had no significant effect on experiential factors in this study (hypothesis 7). The interviews showed that users did not feel much enjoyment during the learning process on the D2D app; they accessed the app with clear goals in mind, such as obtaining the required SKP points or accessing the latest health information:

It’s normal. Enjoy it, I access the app to read discussions, literature, forums.

Respondent 9

Due to the similarity of the features and the flow of the process of using the app, users can become accustomed to or feel bored by it:

It’s actually normal because almost all learning apps have a similar flow, so it’s just normal to me.

Respondent 7

The interviews revealed that users have different perceptions when using mobile learning apps and when using social media apps. They tend to use mobile learning apps for the purpose of learning, whereas social media apps are used for entertainment. Finally, enjoyment related to using the app was found to be influenced by the interactions between users within the app:

In addition, there are still few doctors, and the webinar is very interesting. But the interaction between fellow doctors is lacking.

Respondent 12

The interviews showed that interaction between users is still quite minimal, especially via the discussion feature. To further encourage users to learn, the development team might consider including gamification within the app [13].

#### Hypothesis 8: PHI and Experiential Factors

PHI had no significant effect on experiential factors in this study (hypothesis 8). These results are not in accordance with those of either Alnawas et al [6] or Hsu and Tang [7]; the latter stated that personalization significantly affects experiential factors. Information personalization is important because it connects the app user with the information provided on the app in accordance with the needs and interests of the user [29].

On the basis of the interviews, if the app’s content is in accordance with users’ needs and preferences, it will have an impact on the intensity of use:

From me, it is certain, because it is according to my needs, and I will continue to access it.

Respondent 2

In addition, users with student profiles noted having difficulty finding health information, journals, and webinars aimed at students:

I think it is still lacking because I want D2D to be able to categorize the material. If it can be divided by topic according to the station, it can be a children’s station, so I can use it more comfortably.

Respondent 4

The interviews highlight the disappointment of respondents regarding the suboptimal implementation of personalization. Currently, D2D stores only users’ work profiles, and the app has not optimized content personalization. Finally, it is important to remember that, when implementing personalization, it is necessary to consider the user’s privacy and ensure that previous approval is granted [60].

#### Hypothesis 9: Functional Factors and CARQ

This study showed that functional factors do not have a significant influence on CARQ (hypothesis 9). This is in contrast to the work by Alnawas et al [6]. According to Zhampeissova et al [61], offering too many features or too much information via mobile learning increases the user’s cognitive process load. Therefore, even if the system’s ability to present information is very good, too much variety of that information can distract users from learning [18]. Wilmer et al [62] explained that users’ attention when accessing mobile apps is generally diverted by additional features, resulting in a distraction from the users’ original purpose. Wardaszko and Podgórski [63] stated that the success of cognitive processes is not entirely influenced by the success of the implementation of mobile learning. This is because each individual has different learning styles and cognitive needs, so a given app functionality may be beneficial for certain groups but not for others [63].

In the context of mobile learning, the interviews showed that users accessed the app with a clear purpose of obtaining the latest medical information:

I still need to find a reliable place or platform that can support my learning needs.

Respondent 7

However, users felt that the material provided to support their cognitive needs was unsuitable:

...But unfortunately, it is still rare for information for dentists.

Respondent 3

In addition, it is known that users obtain information in various ways, such as by reading various platforms, by preparing for an examination, and through videos:

To be honest, I cannot learn at this time from only this one platform, because my type of learning has to read as much as possible to understand better.

Respondent 7

Finally, the interviews made clear that users focus more on utilitarian benefits that can be felt directly, such as features that support learning, the collection of SKP points, the quality of the articles and medical information, and the speed of information processing:

Personally, I prioritize the usefulness of the app over the user experience.

Respondent 14

The interviews support that the functionality of the app does not have much effect on the cognitive process because what the user focuses on is the usefulness of the information to support the learning process. This is in line with the work by Cheng [13], who argued that information personalization is a very important part of directing users toward progress in the learning process.

#### Hypothesis 10: Functional Factors and EARQ

This study showed that functional factors affect EARQ (hypothesis 10). Previous research has shown that functional factors affect the emotions felt by users when using retail apps [6]. Furthermore, if the service provider focuses on achieving ease of operation and high performance, this affects the user’s emotions and attitude toward using the app [47]. Chen et al [8] explained that technological capabilities, which implicitly include system performance, have a significant impact on the level of satisfaction with the use of technology in the web-based learning process. Good app performance capabilities, such as information processing speed, can make users feel satisfied and happy because they feel that the app is useful [44]. On the basis of the interviews, the emotions felt by users due to the quality of the app’s functionality significantly affected their decisions regarding continued app use:

...The better the functionality of the app, the more it will meet my expectations, which has an impact on me being happy and likely to keep the app.

Respondent 15

#### Hypothesis 11: Experiential Factors and CARQ

Experiential factors affected CARQ in this study (hypothesis 11). In the context of mobile commerce, user experience has a significant influence on the level of ease of use when operating the app features [8]. Experiential factors are also related to the ease of information collection, which is one of the utilitarian benefits [6]. Dastane and Haba [17] explained that experiential factors can influence a user’s perception of the benefits of the available features either directly or indirectly. Interviewees agreed that the appearance of the app affects the user’s level of trust and their perception of the process of obtaining information:

On the other hand, a good user experience can also make it easier for users to use their features and find information more easily.

Respondent 10

The experience provided by the app also affects the user’s decision to continue using it:

Of course I will move to another one also if it looks bad.

Respondent 9

User considerations in the context of mobile learning still include utilitarianism and the experience provided:

But still in terms of performance, you also have to look at the performance of the app, at least not very slow, it’s still okay for me to use it.

Respondent 11

One of the insights obtained from the interviews is that, if the app provides a suboptimal user experience but the features have a high benefit value, users will still use the platform. However, if a competitor is able to present similar features with a better user experience, users are more likely to switch to them.

#### Hypothesis 12: Experiential Factors and EARQ

Experiential factors were found to affect EARQ in this study (hypothesis 12). Alnawas et al [6] explained that user experience has a significant influence on the relationship between app quality and the emotional judgment of retail app users. Experiential factors also influence the users’ emotions because they provide hedonistic benefits [43]. Improving the user experience is one way to shape the interaction between users and apps; user responses to interacting with an app or service can include emotions, perceptions, preferences, behaviors, and enjoyment [7]. The user experience then affects pleasure during purchasing activities and can encourage users to engage with the app [45]. The interviews showed that a good user experience on the app affects feelings of happiness when using it. Users will also be more interested in using the app if the experience is memorable:

But on the other hand, they will be more interested if the app has good coloring and a cute design.

Respondent 4

In addition, users will also have increased trust in apps that have a good design:

The experience on the app also affects me, so I have more trust in the app.

Respondent 7

#### Hypothesis 13: CARQ and Stickiness in Mobile Learning

This study found that there is a relationship between CARQ and stickiness in mobile learning in health care (hypothesis 13). This result is in accordance with previous research showing that CARQ has a significant influence on stickiness in retail apps [6]. In the context of mobile commerce apps, the results of previous research have shown that the cognitive relationship with apps affects user experience, such as satisfaction levels, and this satisfaction affects the user’s desire to use apps in the future [34,64]. The cognitive relationship dimension includes the ease of searching for information [64] and the ease of efficiently purchasing goods [34]. In addition, CARQ has been proven to have a significant effect on learning persistence in the context of the web-based learning process using massive open online courses [13,65].

The interviews showed that one of the drivers of using the app continuously was users’ desire to obtain the latest medical information:

In my opinion, if from me, the cognitive desire that I usually feel for the learning app is to get new information in the world of health.

Respondent 14

The ability of features to meet user needs also greatly affected the satisfaction that users felt and the intensity of their app use:

So if the app I consider not to provide benefits for me, yes, I also rarely have access intensity.

Respondent 13

However, there were also external factors that affected the intensity of app use, such as busy schedules or poor mobile network quality.

#### Hypothesis 14: EARQ and Stickiness in Mobile Learning

Finally, this study discovered a relationship between EARQ and stickiness in mobile learning (hypothesis 14). In retail app research, it is known that EARQ has a significant effect on user stickiness [6]. The user’s decision to use an app is influenced by the level of emotional satisfaction that they feel [66]. Similar findings were obtained in the study by Souiden et al [67]. In understanding the decision-making process of retail app users, it is important to consider how users feel when shopping in both offline and online stores. In the context of mobile learning, learners’ emotional involvement can positively influence their intention to continue using web-based courses [21]. Zolkepli et al [44] stated that the emotions felt when using an app are affective responses that can be positive or negative and affect the intensity of app use. The emotions felt during app use were found to be related to the user’s desire to continue to use the app regularly:

The feeling I felt while using the D2D app for learning is happy, because the health information provided is quite complete in the app, so it makes me want to continue using the app.

Respondent 15

The sense of pleasure obtained from the experience encouraged users to become more willing to use the app:

I think maybe yes because I’m already happy, so I should at least be willing to use the app.

Respondent 7

## Discussion

### Principal Findings

This study showed that the cognitive and emotional connections with an app affect user stickiness in mobile learning. The user’s EARQ is positively influenced by the performance of the app (functional factors) and the user experience (experiential factors), whereas the CARQ is influenced by user experience factors (experiential factors). App performance can affect emotional assessments triggered by feelings that arise when using apps (hedonic benefits), resulting in subjective assessments. However, the experience that users have when using the app (experiential factors) has an effect on their emotions or feelings. In addition, user experience has an impact on cognitive assessments related to the ease of app use.

This study provides an overview of the influence of the relationship between app relationship quality, namely, EARQ and CARQ, and stickiness in mobile learning. In this study, the organismic variables are EARQ and CARQ, which represent the process of user assessment of app relationship quality [6,13]. EARQ was found to have a greater effect on stickiness in mobile learning apps than CARQ. The emotions felt during the use of an app were found to be related to the user’s desire to continue using the app regularly, and the sense of pleasure obtained from the experience encouraged users to become more dependent on using the app. This is relevant to the results of previous research where, in retail apps, users were more inclined to build affective or emotional-quality relationships than cognitive-quality relationships [6,21]. The results of this study are relevant to the work by Alnawas et al [6] on retail apps, in which the relationship between CARQ and EARQ was found to affect user stickiness. Previous mobile learning research has produced similar results—cognitive and emotional connections were proven to affect users’ desire to continue using apps in the web-based learning process [21]. However, this study showed that functional factors have no effect on CARQ. This is because the app provides diverse information without factoring in users’ information needs.

Mobile learning service providers can use the results of this study to continue to develop features that improve user stickiness in mobile learning. Service providers must pay attention to app quality based on cognitive and emotional aspects when developing features in mobile learning apps. In addition to the usefulness of features, the experience of using the mobile learning app is an important aspect in encouraging ongoing user dependence. Service providers can improve the quality of mobile learning app functionality by focusing on several important aspects, especially the processing speed and quality of the information presented. Processing speed is a critical factor that affects the user experience as responsive and fast apps can improve user satisfaction and efficiency. In addition, the information presented in the mobile learning app must be accurate, relevant, and easily accessible to users. Quality information will help mobile learning app users make better decisions and increase trust in the app. Thus, improvements in these two aspects are expected to encourage user stickiness and ensure sustainable use of mobile learning apps.

Mobile learning service providers can improve the app’s security in the context of personal data protection. To accomplish this, they can present the terms and conditions of the app and privacy policy in a clear and easy-to-understand manner. The purpose of this step is to show the seriousness of the mobile learning service provider regarding safeguarding users’ personal data and building trust with users. In addition, mobile learning service providers can educate users on how to protect their personal data. This education can include instructions not to provide information to unknown parties and on maintaining the confidentiality of personal data.

Furthermore, mobile learning service providers can implement personalized content recommendations and health information tailored to mobile learning users’ interests and information needs. This recommendation feature can be realized by using data on the user’s profession. However, it is important to note that mobile learning user data are confidential and should be protected under the data privacy policy. Important components to consider in the implementation of this feature include effective communication, transparency, engagement, and an attractive value proposition. To increase user enjoyment and interaction during the learning process, mobile learning service providers can improve the gamification features on their apps. This can be accomplished by adopting several game elements, such as missions, rewards, dashboard rankings, and leveling. In addition, gamification provides a good user experience by supporting the learning process and increasing learning persistence.

Because most D2D app users are physicians, there are several external factors that affect the duration of app access. On the basis of the questionnaire, the biggest obstacle was limited time due to a full practice schedule (361/1120, 32.23%). This was also supported by most respondents providing more than one answer related to the location of their practice, the most common being physicians practicing in clinics (267/649, 41.1%), hospitals (117/649, 18%), and private practices (106/649, 16.3%).

### Strength, Limitations, and Future Work

This study enriches previous studies on mobile learning for the Indonesian context using the stimulus-organism-response theory. Another strength of this study is the involvement of medical students, general practitioners, and specialist physicians. However, a limitation of this study is that the research respondents were primarily general practitioners and mobile learning users with an age range of 20 to 30 years; thus, future work should add more specialist respondents. On the basis of the interviews, further research can identify other factors that affect user stickiness, namely, perceived behavioral control and social influence as there were many obstacles users experienced such as busy activity schedules or network quality constraints. In addition, based on the interviews, an in-depth analysis is needed to determine whether encouragement from one’s social environment also affects the desire to continue using an app.

Future work can examine whether there is an influence of moderation factors such as the age, gender, and occupation of mobile learning app users on the relationship between app relationship quality and user stickiness in mobile learning. Future research could also include comparative results based on the age and occupation of mobile learning app users. Further research can explore related factors that affect the app use experience by examining users’ perception of usefulness after learning to use the app.

## Appendix

Multimedia Appendix 1 Research instruments and qualitative results.

## Abbreviations

AVE: average variance extracted
CARQ: cognitive app relationship quality
CME: continuing medical education
D2D: Doctor-to-Doctor
EARQ: emotional app relationship quality
GOF: goodness of fit
HIPS: health information processing speed
HIQ: health information quality
PE: perceived enjoyment
PEU: perceived ease of use
PHI: personalized health information
PPP: perceived privacy protection
SKP: satuan kredit profesional (professional credit unit)

## Data Availability

The data are available upon request from the corresponding authors.
