# Supplementary material for: Health Workers’ Perspectives on Mobile Health Care Learning Stickiness: Mixed Methods Study
Source: JMIR Med Educ. 2025 Jun 13;11:e63827. doi: 10.2196/63827 (PMC12205261; doi:10.2196/63827)
Supplement: Multimedia Appendix 1 [file mededu_v11i1e63827_app1.docx]

**Appendix A. Questionnaire**

| Code | Measurement Items | References |
| --- | --- | --- |
| HIPS1 | I'm satisfied with the m-learning speed responds to my actions. | [1] |
| HIPS2 | I find that the pages in m-learning load quickly. |  |
| HIPS3 | I am happy with the speed of interaction when using the m-learning |  |
| HIPS4 | I find that m-learning make it easy to quickly share information and interact between users |  |
| PPP1 | I believe the m-learning is able to keep my personal data safe | [2] |
| PPP2 | I am confident that my personal data will be well protected by the m-learning service provider | [2,3] |
| PPP3 | I believe that m-learning service providers will not provide my personal data to other parties. | [3] |
| PPP4 | I am sure that the m-learning service provider will keep my personal data confidential well. |  |
| PEU1 | I quickly got used to the various features when I first used the m-learning | [3,4] |
| PEU2 | I don't find it difficult to do what I want in the m-learning | [5] |
| PEU3 | I feel comfortable studying and collecting SKP points in the m-learning. | [3,4] |
| PEU4 | I easily do what I want on the m-learning | [5] |
| HIQ1 | I always get new information about medical studies from the m-learning | [6] |
| HIQ2 | I am confident that m-learning provides accurate and reliable information about medical studies. |  |
| HIQ3 | I believe that m-learning can provide information on medical personnel according to my needs . |  |
| HIQ4 | I believe the m-learning provides relevant information for my work. |  |
| GM1 | I feel driven to achieve the target of collecting SKP points from the m-learning | [7] |
| GM2 | I get a reward in the form of SKP points every time I use the m-learning |  |
| GM3 | I believe that the number of points collected plays a critical role in assessing my level of ability . | [8] |
| GM4 | I feel that there is competition between users in an effort to collect points in the m-learning |  |
| AE1 | I love the look of the m-learning | [1] |
| AE2 | I'm generally impressed with the look of the m-learning |  |
| AE3 | I love the feature layout and the attractively organized look of the m-learning | [5] |
| AE4 | I am happy with the experience of using the m-learning |  |
| PE1 | I am interested in using the m-learning as a forum for learning and communicating related to issues in the medical world. | [9] |
| PE2 | I enjoy the learning process and discussions between users on the m-learning |  |
| PE3 | I find the learning process and discussion on the m-learning fun. | [1] |
| PE4 | I would like to know about all the current information and discussions around medical issues that can be accessed through the m-learning. |  |
| PHI1 | I feel special as a user of the m-learning aimed at medical personnel and medical students. |  |
| PHI2 | I received content that matches my profile as a doctor or medical student | [8] |
| PHI3 | I receive notifications about content that matches my profile and personal needs. | [1] |
| PHI4 | I feel that I have a special space to learn and discuss health issues in the m-learning | [8] |
| FF1 | I believe m-learning can help me meet my job needs well. | [1] |
| FF2 | I believe m-learning has excellent performance. |  |
| FF3 | I believe m-learning rarely experience errors |  |
| FF4 | I believe the m-learning has good quality standards for the content and videos presented. |  |
| EF1 | I feel comfortable using the m-learning to learn and discuss health topics |  |
| EF2 | I feel that the m-learning is a solution to meet the needs of learning in health topics and collecting SKP points. | [10] |
| EF3 | I feel very happy after getting SKP points or reward points from the m-learning | [1] |
| EF4 | I feel satisfied when sharing information or discussing health topics through the m-learning | [10] |
| CA1 | I am satisfied with the m-learning because it helps in collecting SKP points and reading the latest health news. | [1] |
| CA2 | I am happy that the m-learning is in line with my expectations. |  |
| CA3 | I am satisfied with my decision to use m-learning. |  |
| CA4 | I was complacent in the experience of browsing the m-learning | [11] |
| EA1 | I feel less if I don't actively discuss, read health content, or collect SKP points in m-learning | [1] |
| EA2 | I feel connected specifically to the m-learning as a place for medical learning and discussion. |  |
| EA3 | I feel motivated to take part in the medical discussions at m-learning | [6] |
| EA4 | I feel happy using m-learning to access health information. |  |
| SHL1 | I use m-learning every day | [1] |
| SHL2 | I still install m-learning on my smartphone |  |
| SHL3 | I often learn and discuss about health on the m-learning |  |
| SHL4 | I open the m-learning to collect SKP points or read the latest health content. |  |

**Appendix B. Interview Questions**

1. How can the performance of m-learning affect the daily usage rate of the application?
2. How can the influence of the user's sense of dependence (emotion) affect the daily use of m-learning?
3. How does the performance of m-learning affect in meeting user expectations in terms of the ability to provide appropriate information needs (*cognitive app*) and emotional interaction (*emotional app*)?
4. How does the user experience in m-learning affect user expectations regarding app *performance* and emotional *experiences*?
5. Do you think the speed level of m-learning meets your expectations for the functionality (performance) of app?
6. How does the ease of use of m-learning affect the performance or your expectations for the functionality (performance) of app?
7. How can the influence of the quality of information in the application improve the performance or expectations of users on the functionality of m-learning?
8. How does the gamification feature (program points) affect the user experience on the m-learning?
9. How does the visual design of m-learning affect the user experience?
10. How does perceived enjoyment affect the user experience when using the m-learning?
11. How can the influence of the personalized information presented increase the user's desire to continue using the m-learning?

**Appendix C. Qualitative Results**

| **Construct** | **Categorization** | **Frequency** | **Quotations** |
| --- | --- | --- | --- |
| SHL | Flexible access to online learning. | 8 | - - *"... In my opinion, using the app allows for more flexible access, anytime and anywhere..."* **(RK1)**   - *"... It depends on the stasis. I use the app to learn more. As well as when there is time to study and when it is needed"* **(RK4)**   - *"The reason I find something is so easy to find, it looks attractive, it's easy to access anytime and anywhere."* **(RK5)**   - *"The problem is that I use D2D if it's free of charge if there is an online seminar."* **(RK10)**   - *"To collect SKP points, update health information, watch webinars, check (recall) the drug content in the A to Z Drug feature, and read the discussion of the results of doctors on medstalk"* **(RK11)**   - *"To watch webinars and literature"* **(RK12)**   - *"I usually access the application to see discussions and webinars."* **(RK13)**   - *"So far, I've only used the app to view discussions and webinars."* **(RK14)** |
|  | The duration of use of the application < 1 hour a day. | 11 | - - *"I access D2D generally when I am close to the exam can be accessed for about 3-4 hours per day..."* **(RK1)**   - *"If I can do a maximum of 3 hours a day."* **(RK2)**   - *"Honestly, from me per day, it's not much, a maximum of 2 hours, most of the D2D applications that I often check are the medicine part because they make re-calls."* **(RK3)**   - *"The duration is <1 hour, but the base is 2 to 3 times a day to access the application."* **(RK5)**   - *"If the time depends on the needs, it can generally be 1-2 hours to access the application once."* **(RK6)**   - *"I rarely use the D2D application, I can do it 1 - 2 times a week. One access is also usually < 1 hour."* **(RK9)**   - *"I usually have a maximum of < 1 hour of D2D access."* **(RK10)**   - *"I open the application if I need to. Intensity per day < 1 hour. But if you can often access it often, at least 3 times a week to check health information."* **(RK11)** |
|  | The use of the app is only ahead of the exam. |  | - - *The time to use the D2D application is generally when it is close to the exam* **(RK1)**   - *That's why I usually access it if I want class tutorials, plenaries, or exams*. **(RK3)**   - *If I can use a maximum of 3 hours per day.* **(RK2)** |
| CA | Access the application because there is only a need to watch webinars | 6 | - - *"... That's why I access the application only when it's needed."* **(RK1)**   - *"This is because I still have a lot of schedules besides classes, there are practices and other exams. So, access the D2D application if there is a need to study close to the exam or when there is only free time"* **(RK3)** |
|  | D2D applications support a learning process for users |  | - - *"The existence of a learning application for me makes it very easy to memorize, read, skimm, or find the latest news about certain medical cases."* **(RK1)**   - *"Journal and there are information related to the drug glossary that makes it easier for me when studying (re-call) the material."* **(RK2)**   - *"In my opinion, yes, it has an impact, of course, the better the performance of the application, the more comfortable the user will be to use the application***." (RK3)**   - *"I just like the application that is fast and* responsive without having to wait for a long time when *loading."* **(RK4)** |
|  | The features provided are not in accordance with the needs of the user | 4 | - - *"It's just a pity that the job vacancy feature of the locker list presented is not diverse and many are out of date, so it's a bit lazy to access the features, so I really need to look for internship or freelance information"* **(RK1)**   - *"It's just that currently as a student, I haven't used all the features that I often access, the most often I access are Ato Z Medicine, CME (Medical quiz), Medstalk to read, read doctors' discussions, and Journals."* **(RK3)**   - *"So, if I don't think the application will benefit me, yes, I also rarely have access intensity..."* **(RK13)**   - *"In my opinion, because there are many articles that are lacking, there is also a lack of discussion interaction between other colleagues, sometimes I am not satisfied with using the application..."* (**RK12)** |
|  | Access barriers due to a full schedule or network in a weak area. | 5 | - - *"However, because the schedule is quite full of exams or internship preparations"* **(RK2)**   - *"This is because I still have a lot of schedules besides classes, there are practices and other exams. So, access the D2D application if there is a need to study close to the exam or when there is only free time"* **(RK3)**   - *"The only problem is that I have a busy school schedule, so there are barriers to not being able to continue using the application..."* **(RK14)**   - *"The biggest obstacle for me is external factors such as the difficulty of getting a network because I'm a bit in the area, the network likes to disappear."***(RK2)**   - *"... My constraints are from external factors such as the difficulty of getting a network because I am somewhat in the region..."* **(RK15)** |
|  | The need to learn from the app is influenced by the latest medical information. | 3 | - - *It's different if you learn through a website, for example, the tabs have been opened a lot later, so it's a bit slow to load, if it's in the D2D application, it's very fast to download journals and watch webinars.* **(RK1)**   - *"I think yes, if from me, it's usually cognitive that I feel for a learning application to get new information in the world of health, yes..."* **(RK14)**   - *"Some of the factors that support the information presented are appropriate and interesting not, the offer of SKP point needs, and discussion..."* **(RK13)** |
| EA | There is an encouragement to use applications if they have a good experience such as gamification and notifications | 5 | - - *"On the other hand, because of the notification, it helps me to trigger it as well if there is a reminder to access the application."* **(RK1)**   - *"I think as long as the user feels happy or enjoys using the application, it will certainly affect the sense of dependence to continue to take care of the application..."* **(RK3)**   - *This is because there is no need to learn* **(RK4)**   - *"I think maybe it's because I'm already happy, so yes, I'm at least willing to use the application."* **(RK7)**   - *"In my opinion, the more the application is easier to use and comfortable to use, it must be something easy, so I will continue to install the application because it suits my needs. My mindset is that if you need something about the medical world, just use a D2D application. The tendency to use applications is also not consciously remembered to use D2D applications"* **(RK6)** |
|  | Learning from apps is more practical | 2 | - - *"I think learning through D2D applications feels more practical."* **(RK1)**   - *"And that's why it's so important for you to learn from yourself."***(RK4)** |
|  | Learning through the app is only close to the exam | 2 | - - *"I think so, because I become quite dependent on studying through the D2D application if it is indeed close to the exam"* **(RK1)**   - *"But it needs to be noted that I don't use the application every day, but it depends on my needs."* **(RK4)** |
|  | The user's feelings affect stickiness | 5 | - - *"The feeling I feel while using the D2D application to learn is happy because the health information provided is quite complete in the application"* **(RK2)**   - *"Of course, it doesn't have an effect, if from my own perspective the more comfortable I am using the application, of course I will be happy to continue using the application."* **(RK2)**   - *"I feel like it's more fun because there is an app that facilitates being able to see the opinions of doctors' discussions"* **(RK3)**   - *"In my opinion, as long as the user feels happy or enjoys using the application, it will certainly affect the sense of dependence to continue using the application"* **(RK3)**   - *"The feeling I felt while using the D2D application to study was happy, because the health information provided was quite complete in the application, so I had the desire to continue using the application..."* (RK15) |
| FF | Good app performance and *fast* loading | 6 | - - *"So far, I have never found any errors or errors"* **(RK1)**   - *"As long as I've been using it now, I've never found any errors."* **(RK2)**   - *"In my opinion, the D2D application has good performance. Honestly, it loads very fast and I really like it"* **(RK1)**   - *"In terms of application functionality, the D2D application has been able to meet my expectations in terms of information provision ability, loading speed, and minimal errors, which is okay"* **(RK1)**   - *"I use it smoothly; the loading is also fast and the webinar video is also smoothly played. At least if it's a bit delayed when watching the webinar, it's because of the network constraints if I do."* **(RK2)**   - *"D2D is good, it doesn't take too long to open the application. The loading page is very fast."* **(RK3)** |
|  | The information provided does not match the user's persona | 5 | - - *"But it's unfortunate for me as an internship doctor that the information in journals or webinars is provided more to specialist doctors, I ask that maybe it can be multiplied with content whose information can be used for general practitioners as well."* **(RK1)**   - *"My expectations are related to the assessment of the application that the application is able to meet the needs of users, especially the presentation of information"* **(RK2)**   - *"I still need to find a trusted place or platform that can support my learning needs"* **(RK7)**   - *"But it's unfortunate for me as an internship doctor that the journal or webinar information provided is more for specialist doctors, I ask that maybe we can just increase the content whose information can be used for general practitioners as well."* **(RK1)**   - *"... But unfortunately, it is still rare for information for dentists"* **(RK3)** |
|  | The effect of app performance on user cognitive | 6 | - - *"Of course, it will affect if the better the functionality of the application, it doesn't crash or when using the features smoothly, of course, it will definitely meet my expectations regarding the assessment of the application that the application is able to meet the needs of users, especially the presentation of information."* **(RK2)**   - *"so, it further affects the ease of users to access the features"* **(RK3)**   - *"I think the quality of the functionality of the app definitely directly affects my performance rating to the app."* **(RK4)**   - *"Then from me to be honest, to learn now is not only from 1 platform, because my type of learning has to read as much as possible to understand better"* **(RK7)**   - *"... On the other hand, I also have access to the learning application that is easier to approach the exam, because of the concise presentation of information to repeat the learning material."* **(RK9)**   - *"I honestly prefer to learn through GT webinars, because it saves time and usually the information is dense GT compared to offline training"* **(RK12)** |
|  | App performance affects user experience | 6 | - - *"... happy and likely will still install the application."* **(RK2)**   - *"On the other hand, if the application works well and has minimal errors, it will certainly make me happy and comfortable when accessing the application."* **(RK3)**   - *"And if it's a good fit, I'm happy with it."* **(RK4)**   - *"When there is new content, I can still see it through notifications on my phone. This makes the user experience more practical and makes it easier for me to stay connected with the latest information."* **(RK1)**   - *"If the functionality is not good, yes, the impact will be that I get annoyed, how can the application be bad..."* **(RK12)**   - *"... the better the functionality of the application, the more it will meet my expectations which has an impact, I will be happy and likely to continue to install the application"* **(RK15)**   - *"It's just that if the performance is good, but I don't feel happy with the interaction experience with the application, I get lazy to use the application, and the intensity of using the application is also somewhat reduced."* **(RK11)** |
| EF | The influence of user experience on cognition can be in terms of trust, satisfaction, and usefulness felt by users. | 10 | - - *"Too much information is displayed, and the language selection is difficult to understand, or the application turns out to be often error-prone so I am lazy to use the application."* **(RK2)**   - *"The more I get a good user experience, the easier and more satisfied I will be to use the application."* **(RK3)**   - *"But on the other hand, you will be more interested if the application has good coloring and a funny design. The impact of the emotions that I felt was certainly happy because the application was beautiful to look at. Another thing I feel is that it makes me feel more comfortable using the app."* **(RK4)**   - *"I think the design, layout, color selection of the application can affect the level of ease I use the application"* **(RK1)**   - *"I think in terms of design, the appearance is already good, the color selection is also interesting."* **(RK2)**   - *"But on the other hand, you will be more interested if the application has good coloring and a funny design. The impact of the emotions that I felt was certainly happy because the application was beautiful to look at. "I think it's going to be a lot easier for me to use the app."*   - *"On the other hand, a good user experience can also make it easier for users to use the features and find the information more easily"* **(RK10)**   - *"The application looks good. The flow is easy to understand, and I also find it easy to find information in it"* **(RK8)**   - *"This is also even though the experience is bad, but the features offered are good, whether you like it or not, you will still use it. It's just that if there are competitors who offer the same features and a better experience, I'd rather move on."* **(RK14)**   - *"But still in terms of performance, you also have to see from the performance of the application, at least it's not very slow, it's still okay for me to use it"* **(RK11)** |
|  | EF's influence on user experience | 8 | - - *"It's very influential. because from me, for example, even though it has been recommended by people and they say it's good, but when I tried it, it turned out that the design wasn't good, like the layout was ugly, the color selection made the eyes hurt, the font writing was unreadable"* **(RK2)**   - *"Conversely, if for example the UI/UX is bad, yes, I am also lazy to use the application and I can even uninstall it."* **(RK3)**   - *"But on the other hand, you will be more interested if the application has good coloring and a funny design. The impact of the emotions that I felt was certainly happy because the application was beautiful to look at. Another thing I feel is that it makes me feel more comfortable using the app."* **(RK4)**   - "*I will also like to use the application and want to continue to access the application if the experience is good, both from the choice of colors, fonts, rewards and some of it is what makes it exciting to use the application."* (**RK5)**   - *"Then the experience on the application has affected me so I trust the application more"* **(RK7)**   - *"But on the other hand, you'll be more interested if the app has good coloring and a funny design."* **(RK4)**   - *"... Because from me, for example, even though it has been recommended by people and they say it's good, but when I tried it, it turned out that the design wasn't good, like the layout was bad, the color selection was a pain in the eye, the font writing was unreadable, too much information was displayed, and the language selection was difficult to understand, or the application turned out to be often error-prone, so I was lazy to use the application."* **(RK15)**   - *"... If it's a good idea, then it's a bad idea, but I don’t think it's a good idea."***(RK6)** |
| HIPS | The speed of information processing in the application is good. | 4 | - - *"It's very good, this has also met my expectations for the functionality of the D2D application"* **(RK2)**   - *"The application* response *speed is very good and fast"* **(RK1)**   - *"As long as I've been using it, I've never experienced a failure to load on the application page or a gt error."* **(RK2)**   - *"If I think the loading is good, it's not too bad that it has appeared for a while"* **(RK3)** |
|  | Network constraints in poor areas | 1 | - - *"The most indeed what I have said is that the network in my area is bad. That's why I access the application if there is a good WiFi."* **(RK2)** |
|  | Better speed of information than competitors | 2 | - - *"Of course, it's a lot of pressure like I used to use Umeds, actually it's good to have animation but the problem is that the material is very long"* **(RK3)**   - *"I think this is very good compared to when I opened a journal website or an application from a competitor (Alom*dika)*" **(RK1)** |
| PPP | The problem is that users often forget their accounts and passwords | 2 | - - *"On the other hand, I know that to maintain the security of user data, sometimes we have to log in again and enter emails and passwords. The problem I experienced was that I often forgot my account or password and it took time to reset the password again****.*** *(* **RK1)**   - *"... If I use the application, I often forget the account name or password."* **(RK2)** |
|  | Users' beliefs about the extent to which their personal information and data are protected by the App do not affect the app's functionality assessment | 4 | - - *"I think applications that have good security can use PIN, fingerprints, etc. So far, but I don't fully believe in D2D, actually, because I haven't entered my personal data in the D2D application because I don't have STR and SIP, my current status as a medical student."* **(RK15)**   - *"I don't think it has much effect. Since the data is not secure, I hesitate to use the app. My personal data is not being used for what it should be. Honestly, the functionality is more about the process of creating the password and verifying it is not complicated and makes it easier for the user. "If you want to build trust in the user, then you have to trust the user."***(RK13)**   - *"I don't think there is. This is because the performance of the application (functionality) is more inclined to the ability of the features in the application to run well, such as loading, information provided, and minimal errors. While the level of security in my opinion is more to the standard of the application, it must have good security, and this is not too close to the functionality of the feature in my opinion."* **(RK11)**   - *"As a student, I don't think it affects too much, because the main function of the application is to support the process of using the application to learn so that the content or information presented is relevant or not"* **(RK14)** |
|  | Users trust applications that have collaborated with trusted organizations | 3 | - - *"That's why my efforts to give personal data to the application usually check first whether the application has collaborated with trusted organizations such as IDI, the Ministry of Health, and KOMINFO."* **(RK2)**   - *"If I am personally a gen Z, it doesn't really affect me too much. So far, for my purposes, it's okay. As long as the application has partnered with trusted organizations, there will continue to be TnC and a privacy policy as well."* **(RK8)**   - *"I don't think this is so much an influence between security and feature performance, because the two things should be regulated separately. Where performance is more about information output, while security focuses on building user trust in the application"* **(RK12)** |
|  | The importance of STR and SIP data for Doctors | 3 | - - *"To be honest, I'm actually a bit scared to share STR and SIP documents to the application. The reason is that the application can be an error or it can be hacked, right"* **(RK2)**   - *"... and must enter personal data such as STR and SIP numbers without any explanation of what the need is for."* **(RK2)**   - *"If there is currently as much SIP, I will love STR, because this is indeed a doctor's learning application, right? For example, the phone number should be optional."* **(RK3)** |
|  | The importance of the role of the service policy information page and the user privacy policy. | 4 | - - *"On the other hand, the thing that I consider in registering an account in the application is the existence of a TnC page that clearly and in detail explains that this application guarantees the security of user data by not disseminating it to any party."* **(RK1)**   - *"I think D2D applications already have a good security system judging by the clear and detailed TnC about keeping user data safe*."**(RK1)**   - *"The reason is that he asked for information on the terms and conditions as well, so I quite trusted the application*." **(RK3)**   - *"If I am personally a gen Z, it doesn't really affect me too much. So far, for my purposes, it's okay. As long as the application has partnered with trusted organizations, there will continue to be TnC and a privacy policy as well."* **(RK8)** |
|  | The Importance of Friend Recommendations or *word-of-mouth* (WoM) | 2 | - - *"In addition, word-of-mouth or recommendations from people/friends/colleagues are also very influential in increasing my trust in the application."* **(RK2)**   - *"Maybe from my colleague who makes, Word-of-mouth, the information given should have an explanation of what the information is for."* **(RK3)** |
|  | Too long registration and verification process | 6 | - - *"To be honest, the thing that sometimes makes me lazy to use the application is the long registration process, it needs a lot of verification as well"* **(RK2)**   - *"Sometimes when there are too many things that need to be filled, I'm lazy to continue to register."* **(RK2)**   - *"I don't think it has much effect. If you ask me if I don't want to use the app, I'm hesitant to use it. My personal data is not being used for what it should be. Honestly, the functionality is more about the process of creating the password and verifying it is not complicated and makes it easier for the user. "If you want to build trust in the user, then you have to trust the user."* **(RK13)**   - *"Honestly, I also feel that the login and registration process is a bit long. It's nice to have a login preference by google, but I'm a bit lazy because I still have to fill in my personal data and medical student number etc. Honestly, I'm a bit lazy to give and reluctant to give because so far I haven't had the need to collect SKP points, if it's just for study or case study, it's better to just look for a website."* **(RK7)**   - *"What data is requested first, if D2D asks for the doctor's registration number? Another difference is that what is asked is an ID card number or a photo with my new ID card. I also think that the registration and verification process is fast which affects the functionality of the system or application"* **(RK6)**   - *"In my opinion, yes, actually, the more people are asked for, the better. But, on the other hand, I'm pretty damn lazy. Applications, for example, are not too important, so I will not enter them and prefer not to use them"* **(RK4)** |
|  | Too much personal data requested | 2 | - - Actually, one of the things that makes me a bit lazy to use the application is the amount of information that must be included such as STR number, SIP number, email, cellphone number and so on **(RK1)**   - If there are currently as many SIPs, I will love STR, because this is indeed a doctor's learning application, right? For example, the phone number should be optional. **(RK3)** |
| LITTLE | Selection of feature names and elusive form titles | 2 | - - *"There are only a lot of feature names that I don't understand, for example, P2KB albums turn out to be a list of SKP points and certificates"* **(RK2)**   - *"In addition, it is also explained how to form for users who are doctors and medical students as well, because I myself already have an STR but for internships and SIP yet."* **(RK2)** |
|  | Limited access to features and literature for medical students | 3 | - - *"If I have more difficulty because I can't verify IDI because I'm a student and the content I still have access to is limited"* **(RK2)**   - *"I'm not too satisfied with myself because I haven't been able to explore many things huhuhuhuh:( I'm still not a doctor"* **(RK3)**   - *"Actually, this feature is complete, it's just that I'm very unfortunate because of FKG, now there is no literature that is familiar to dentists, why is there nothing that I need. Maybe it can be added to the status of FKG."* **(RK8)** |
|  | Users have difficulty operating the application both during login, verification process, and registration | 5 | - - *"I have an obstacle when I have to enter the STR Number. Actually, I haven't gotten STR yet, so I'm a bit hesitant because I haven't gotten STR because it's still an internship status."* **(RK13)**   - *"I get into trouble quite often. Suddenly came out and went in again. Suddenly came out and went in again. Entering the NPA IDI again is a shambles"* **(RK12)**   - *"To be honest, I was confused at the beginning of the registration, I think the information from the title field is not clear. For example, there is a part of the form that asks for a medical id card, so there is no information at the beginning of the medical id card what it is like whether it is STR, SIP, NIM or what."* **(RK2)**   - *"Confused, often sudden updates"* **(RK10)**   - *"If I have more difficulty because I can't verify IDI because I'm a student mash and the content I still have access to is limited"* **(RK3)** |
| HIQ | The quality of complete information in the application can minimize misunderstandings, interpretations of information or the spread of information that is false (*hoax).* | 2 | - - *"The existence of good information quality is aimed at minimizing the misunderstanding of hoax information because the source of information is not detailed and clear."* **(RK2)**   - *"It is influential, of course, because with the information presented that is accurate and credible, as a user I also trust and feel useful for learning in the application. On the other hand, it also minimizes the misunderstanding of hoax information because the source of information is not detailed and clear."* **(RK15)** |
|  | The quality of information also encourages users to recommend the application to their peers | 4 | - - *"Yes, it really affects. Even if for example it is complete, I can also recommend it to friends"* **(RK4)**   - *"I think it has something to do with it, if he can give good reliable information, of course there will be many people who will use it later"* **(RK9, RK12, RK13)** |
|  | The quality of accurate and reliable (credible) Health Information significantly affects motivation and assessment of good application functionality. | 6 | - - *"With accurate information, users will feel more comfortable using the application. For example, when there are complete and easily accessible references, such as direct links to journal website pages, or the availability of complete webinar recordings with well-known speakers and the existence of official certificates issued in the application, it increases my motivation to continue learning using the D2D application."* **(RK1)**   - *"Yes, because honestly this is what we need the most as students because the focus is on learning. This really interests me."* **(RK3)**   - *"It has something to do with it in my opinion. So if the information provided is accurate, it will be a good assessment for the performance of the application. (***RK5, RK6, RK7, RK8)** |
|  | The quality of information affects the level of trust and usefulness of users | 6 | - - *"In my opinion, it has a big impact on the user's level of trust in the performance of the application, because with accurate information, the user will feel more comfortable using the application"* **(RK1)**   - *"It is influential, of course, because with the information presented is accurate and credible, as a user I also trust to learn in the application."* **(RK2)**   - *"I need to be a student because it's really focused on learning."* **(RK3)**   - *"Yes, because it's an application for learning. If the information is not good, then why am I there to study"* **(RK10)**   - *"I think this is very influential because performance is also measured by how well the application is able to provide reliable information for users. Because the application is intended for doctors and learning, it must also be good and feel useful when using the application"* **(RK11)**   - *"It is influential, of course, because with the information presented that is accurate and credible, as a user I also trust and feel useful for learning in the application. On the other hand, it also minimizes the misunderstanding of hoax information because the source of information is not detailed and clear."* **(RK15)** |
| GM | The adoption of gamification in the collection of SKP points encourages motivation and a sense of competitiveness for users. | 3 | - - *"Because there are rewards, and there is a mission to be given, so I feel more competitive to use the application"* **(RK2)**   - *"... I feel like I'm getting more competitive with the app"* **(RK15)**   - *"D2D is more attractive than competitors for its gamification features. The dashboard is also interactive and informative"* **(RK11)** |
|  | Gamification encourages the learning process | 5 | - - *"It feels like a learning application with a gamification feature makes it exciting and exciting."* **(RK1)**   - *"It is precisely because there is a challenge or mission that I am motivated to learn in the D2D application."* **(RK2)**   - *"There is an explanation that CME, there are SKP points and watch webinars so I can access them at any time. Especially here the SKP points are also big, these 2 SKP must be very interesting because it is easier to be able to cash at any time"* **(RK3)**   - *"I think this is one of the main factors for using D2D. Unlike the application next to only track records the number of SKP points, it has not been able to collect SKP points"* **(RK6)**   - *"I think there is a target and I feel more appreciated if I watch webinars or learn in D2D applications"* **(RK1)** |
|  | The adoption of gamification for the collection of SKP points cannot be used by medical students because they do not have a Registration Certificate (STR) | 3 | - - *"Never made it, because there is no need yet"* **(RK3)**   - *"Currently, I have never used it because there is no need and I can't either. I'm still a 2nd year medical student"* **(RK5)**   - *"Currently, there is no need to use SKP points because I am still using STR Internship"* **(RK9)** |
| Yes | If the app's visuals are engaging, it will encourage the continued use of the app | 3 | - - *"I think the appearance of the (visual) design of the application has a great influence on the level of application usage."* **(RK1)**   - *If the app looks attractive, is easy to use, and the selection of icons is attractive, users will feel comfortable using the app continuously"* **(RK1)**   - *"I think it's very important, if from the beginning it's not interesting I'll be lazy to open it again"* **(RK5)** |
|  | The color selection should be inconspicuous | 4 | - - *"The choice of colors that are not too flashy makes me more comfortable when using the D2D application"* **(RK1)**   - *"And the design is also always updated so that it doesn't get boring and doesn't use bright colors"* **(RK2)**   - *"On the other hand, the choice of color can also be found as an alternative not to the one that is cold"* **(RK13)**   - "I think it *might be possible to improve the color of the design not all the red so that there is a white gt so that it doesn't be flashy"* **(RK15)** |
|  | The importance of the text size and feature layout in the app | 4 | - - *I think the writing is too small, so it's a bit difficult to read"* **(RK12)**   - *"The selection of text sizes is too small"* **(RK15)**   - *"Most of the time in the journal section, the name of the journal doesn't look full, so it's a bit difficult"* **(RK7)**   - *"On the other hand, the layout of the features is not confusing, so I feel that the D2D app already has an ideal visual appearance."* **(RK1)** |
|  | The design display is less interactive and rarely *updated*. | 5 | - - *"And the design is also always updated so that it doesn't get boring and doesn't use bright colors"*  **(RK2)**   - *"... The design is also always updated so that it doesn't get boring"* **(RK15)**   - *"If possible, the excellent features that are emphasized in the medical discussion so that it can be more interactive between users"* **(RK12)**   - *"My suggestion is that I can add a mascot or other animation to make it interesting"* **(RK5)**   - *"But my suggestion may be given other interactive elements, for example there is a moving mascot"* **(RK13)** |
| PE | Medical students tend to face difficulties when studying on applications because they often do not find content that suits the needs of users. | 3 | - - *"On the one hand I don't feel too happy (excited) but on the other hand I'm not disappointed either, so yes neutral"* **(RK1)**   - *"For example, an application for learning is because there is a need but it is not used too often because the information is generally used if I just want to learn. Because of my needs, I want very specific data. I also need a lot of journals related to case studies."* **(RK4)**   - *"To be honest, I still don't really enjoy it because it's a new user too. Because there are no requirements for collecting SKP points. Second, you check that there are rarely topics for dentistry. Disclaimer doctors and dentists are different topics. So I feel like I'm still limited to using D2D applications."* **(RK8)** |
|  | The main purpose of application access is only to learn so that there is coercion that is different from social media applications which are *entertaining*. | 6 | - - *"So my impression every time I open the D2D application is that I want to learn to increase knowledge, be it reading journals, filling out quizzes, watching webinars, and finding information quickly through mobile applications."* **(RK1)**   - *"It's really nice to be able to do that because there's a lot of pressure to continue learning anytime****."* (RK1)**   - *"In my opinion, if it is for learning applications, because there are indeed guidelines to get information or learn new things, it feels less enjoyable***" (RK1)**   - *"I'm really not happy because this is just a demand to continue learning. Now with a D2D application that is always update via notifications. And fortunately, the webinar presentation was interesting and didn't make a fuss so I'm excited if the webinar topic is interesting***" (RK10)**   - *"It's normal actually, because almost all learning applications have the same flow similarity, so from me, it's just normal. And I think it’s important for me to be able to learn from my peers"* **(RK7).**   - *"It's just normal, actually. I don't have a lot of time to read discussions, literature, forums. Keep it open if you are free or there is an interesting notification"* **(RK9)** |
|  | The lack of interaction between users and the similarity of features with competitors causes users to become less interested in using the application. | 5 | - - *"But I still don't dare to join the discussion, so yes, currently I am still reading the case study discussion between doctors. To be honest, if it's urgent, I personally prefer to ask my peers because they know me better."* **(RK3)**   - *"It's normal actually, because almost all learning applications have the same flow similarity, so it's just normal for me."* **(RK7)**   - *"In my opinion, yes because even though there are many competitors from mobile learning D2D applications, there may be subjectivity based on the offering of features (top features) of advantages that are remembered by users. So he knows what the branding of this application is, so this is what can affect the enjoyment and assessment of a good experience from users to the application"* **(RK8)**   - *"Yes, it's nice to get new insights from several other doctors. But yes, to be honest, it's still lacking because it needs access to reputable journals as well, there are still few users who are active in discussions, so it's less interesting. Maybe there can be a program to be able to activate the discussion to get points or vouchers"* **(RK9)**   - *"I think it's normal. The reason is because the writing is a bit too small in my opinion. In addition, there are still few doctors and the webinars are very interesting. However, the interaction with fellow doctors is lacking. The webinar was fun and fun. Then the discussion is also not active in D2D. The doctor is still passive, maybe we can focus on activating discussions on the D2D platform."* **(RK12)** |
|  | Users access the app only when needed. | 4 | - - *"It's like I open a D2D application if it's exam season or there is a need to learn something."* **(RK1)**   - *"The only thing that I don't enjoy is sometimes the demands from lecturers or doctors to look for information sourced from well-known journals."* **(RK2)**   - "*Indeed, this is the focus for learning, so yes, happy or unhappy. Because of the access, I usually take 3-4 hours at most. But I don't wear it every day"* **(RK4)**   - "*I don't think it's too much, it's more about the content if it meets my needs to learn, right? I think the user experience is good."* **(RK7)** |
| PHI | The information presented on the application is less specific according to the user's needs. | 3 | - - *"... The information presented is less specific or it can be said that it cannot yet include the suitability of the user persona..."* **(RK1)**   - *"... Because of this, in my opinion, in D2D, the number of webinars is not interesting for doctors, internships are not yet according to my needs."* **(RK2)**   - *"... Less than me. My expectation is that I want more of the same literature to be our preference for writing and discussion"* **(RK3)** |
|  | Lack of content or information for general practitioners or internships. | 4 | - - *"It's just that sometimes I want content that really suits my persona such as the basic things in the emergency room and content that is more general in nature that can be consumed by students as well****."* (RK1)**   - *"In my opinion, it's still lacking and rare for my persona as a medical student or an intern doctor."* **(RK2)**   - *"I think it's still too common, in my opinion. I expect that I have suggestions, information, guidelines to handle in the emergency room or primary services."* **(RK9)**   - *"To be honest, I can't be a medical student. Maybe I need more options to enter STR, I think it's also a bus to enter the screening or guideline so that it's interesting and helps me also to continue using the application. D2D applications will be very useful..."* **(RK13)** |
|  | Users are likely to continue using the app if the content presented is in line with their needs and preferences. | 4 | - - *"In my opinion, even though the information has been personalized, it will be an advantage, but for me, it is somewhat adjusted to the intensity of using the application because of the fairly tight schedule. But I'm still going to use it because it's a way for me to learn more easily"* **(RK1)**   - *"If it's from me, it's definitely yes, because it's according to my needs and I'll continue to access it"* **(RK2)**   - *"To be honest, if for example, the journal is multiplied by literature, it always updates every week, yes, I will access the application. Because it makes it easier for me to access anytime and anywhere"* **(RK3)**   - *"Yes, of course, actually, because from the student side I actually need it to look for journals and literature. It would be very useful for me to display the latest journals"* **(RK4)**   - "*I expect that I have suggestions, information, guidelines to handle in the emergency room or primary services."* **(RK9)**   - *"I think it's going to be a good thing, and I'm going to continue to do that."***(RK14)** |
|  | Limited access to features in the D2D application for Medical Students. | 5 | - - *"In my opinion, it's still lacking and rare for my persona as a medical student or an intern doctor. Then also the data presented is not my needs. The title of the webinar and the literature provided are still not in accordance with what is needed, because I am still a student and I need webinars such as handling diseases that are often in primary care or emergency departments."* **(RK2)**   - *"It's less than me, my expectations are that I want more of the same literature to be our preference for writing and discussion. The webinar has not been able to participate because it is now a student's status."* **(RK3)**   - *"In my opinion, it is still lacking because I want to be able to categorize the material in D2D. If it can be divided per topic according to the stasis, it can be a child or a pulmo so I feel more comfortable and use it."* **(RK4)**   - *"I feel really bad for FKG students, to be honest, it still seems like it is still prioritized only for medical students. So, I feel a little bit limited. Most of the time, use the application to check the list of A-Z drugs and read interesting discussions in the medstalk feature. From the application side, this webinar is really good, actually, if the doctor who makes it has been registered with SKP points."* **(RK8)**   - *"From my point of view, I am also a bit disappointed because many features are limited access because I am still a student."* **(RK10)** |

**References**

1. Alnawas I, Al Khateeb A, El Hedhli K. The effects of app-related factors on app stickiness: The role of cognitive and emotional app relationship quality. Journal of Retailing and Consumer Services 2023;75:103412. doi:10.1016/j.jretconser.2023.103412
2. Chen YH, Chien SH, Wu JJ, Tsai PY. Impact of signals and experience on trust and trusting behavior. Cyberpsychology, Behavior, and Social Networking 2010;13(5):539-546. doi:10.1089/cyber.2009.0188
3. Kim MJ, Lee C-K, Jung T. Exploring consumer behavior in virtual reality tourism using an extended stimulus-organism-response model. Journal of Travel Research 2018;59(1):69-89. doi:10.1177/0047287518818915
4. Lu Y, Wang B, Lu Y. Understanding key drivers of MOOC satisfaction and continuance intention to use. Journal of Electronic Commerce Research 2019;20(2):105-117.
5. Huang Y-C, Chang L-L, Yu C-P, Chen JS. Examining an extended technology acceptance model with experience construct on hotel consumers’ adoption of mobile applications. Journal of Hospitality Marketing & Management 2019;28:957-980. https://api.semanticscholar.org/CorpusID:169303057
6. Elsotouhy MM, Ghonim MA, Alasker TH, Khashan MA. Investigating health and fitness app users’ stickiness, WOM, and continuance intention using S-O-R model: The moderating role of health consciousness. International Journal of Human-Computer Interaction 2022;40(5):1235–1250. doi:10.1080/10447318.2022.2135813
7. Yang D, Li C. Design of gamification theory in tourism application: Take the application “Travel in Zhenjiang” for example. 2020 International Conference on Innovation Design and Digital Technology (ICIDDT); 2020. 290-294. doi:10.1109/ICIDDT52279.2020.00059
8. Cheng YM. What makes learners enhance learning outcomes in MOOCs? Exploring the roles of gamification and personalization. Interactive Technology and Smart Education 2023. doi:10.1108/ITSE-05-2023-0097
9. Lin PY, Liang TP, Huang HC, Li YW. Design Quality, Relationship Intimacy and Continuance Intention of Mobile Apps: an Extension To the Is Success Model. Journal of Electronic Commerce Research 2021;22(4):266–284.
10. Shahid S, Islam JU, Malik S, Hasan U. Examining consumer experience in using m-banking apps: A study of its antecedents and outcomes. Journal of Retailing and Consumer Services 2022;65:102870. doi:10.1016/j.jretconser.2021.102870
11. Molinillo S, Aguilar-Illescas R, Anaya-Sánchez R, Carvajal-Trujillo E. The customer retail app experience: Implications for customer loyalty. Journal of Retailing and Consumer Services 2022;65:102842. doi:10.1016/j.jretconser.2021.102842
